# Epidural Spinal Cord Stimulation for Spinal Cord Injury in Humans: A Systematic Review

**DOI:** 10.3390/jcm13041090

**Published:** 2024-02-14

**Authors:** J. I. Chalif, V. S. Chavarro, E. Mensah, B. Johnston, D. P. Fields, E. J. Chalif, M. Chiang, O. Sutton, R. Yong, R. Trumbower, Y. Lu

**Affiliations:** 1 Department of Neurosurgery, Brigham and Women’s Hospital, Boston, MA 02115, USA; jchalif@bwh.harvard.edu (J.I.C.); vchavarro@mgh.harvard.edu (V.S.C.); bjohnston2@mgb.org (B.J.);; 2 Harvard Medical School, Boston, MA 02115, USA; mchiang@mgh.harvard.edu (M.C.); ryong@bwh.harvard.edu (R.Y.); rtrumbower@mgb.org (R.T.); 3 Department of Physical Medicine and Rehabilitation, Spaulding Hospital Cambridge, Cambridge, MA 02115, USA; 4 Chan School of Public Health, Harvard University, Boston, MA 02115, USA; emensah@hsph.harvard.edu; 5 Department of Neurological Surgery, University of Pittsburgh, Pittsburgh, PA 15261, USA; fieldsdp@upmc.edu; 6 Department of Anesthesiology Perioperative and Pain Management, Brigham and Women’s Hospital, Boston, MA 02115, USA; osutton@bwh.harvard.edu

**Keywords:** epidural spinal cord stimulation, spinal cord injury, sensorimotor function, autonomic function

## Abstract

(1) Background: Spinal cord injury (SCI) represents a major health challenge, often leading to significant and permanent sensorimotor and autonomic dysfunctions. This study reviews the evolving role of epidural spinal cord stimulation (eSCS) in treating chronic SCI, focusing on its efficacy and safety. The objective was to analyze how eSCS contributes to the recovery of neurological functions in SCI patients. (2) Methods: We utilized the PRISMA guidelines and performed a comprehensive search across MEDLINE/PubMed, Embase, Web of Science, and IEEE Xplore databases up until September 2023. We identified studies relevant to eSCS in SCI and extracted assessments of locomotor, cardiovascular, pulmonary, and genitourinary functions. (3) Results: A total of 64 studies encompassing 306 patients were identified. Studies investigated various stimulation devices, parameters, and rehabilitation methods. Results indicated significant improvements in motor function: 44% of patients achieved assisted or independent stepping or standing; 87% showed enhanced muscle activity; 65% experienced faster walking speeds; and 80% improved in overground walking. Additionally, eSCS led to better autonomic function, evidenced by improvements in bladder and sexual functions, airway pressures, and bowel movements. Notable adverse effects included device migration, infections, and post-implant autonomic dysreflexia, although these were infrequent. (4) Conclusion: Epidural spinal cord stimulation is emerging as an effective and generally safe treatment for chronic SCI, particularly when combined with intensive physical rehabilitation. Future research on standardized stimulation parameters and well-defined therapy regimens will optimize benefits for specific patient populations.

## 1. Introduction

Spinal cord injury (SCI) is a catastrophic complication of trauma which may result in the loss of sensorimotor and/or autonomic function. With an annual incidence of 18,000 people and an estimated prevalence of 368,000 people in the United States [1], SCI places a significant burden on the overall health and economy of the country [1,2]. Commonly affecting younger individuals, the total estimated lifetime cost of care for an injured individual aged 25 ranges from $1.6 million to greater than $5 million [1].

Severity of SCI is graded according to the American Spinal Injury Association (ASIA) Impairment Scale (AIS): AIS-A is clinically complete motor and sensory SCI patients (no muscle power and sensory function); AIS-B is motor complete and sensory incomplete; AIS-C is motor incomplete with >50% of muscles below the neurological level with a muscle grade <3; AIS-D is motor incomplete with ≥50% of muscles below the neurological level with a muscle grade ≥3; and AIS-E is normal [3]. In addition to these sensorimotor deficits as defined by the ASIA Impairment Scale, SCI may also produce secondary autonomic complications, including cardiovascular, gastrointestinal, genitourinary, or respiratory dysfunction [4].

Current treatment for SCI and its associated complications is multidisciplinary and includes a combination of medical, surgical, and neuro-rehabilitation strategies [5,6]. Despite these approaches, the functional improvement after SCI typically plateaus, necessitating adaptive strategies for activities of daily living [7]. Research into innovative assistive technologies [8,9], cell replacement therapy [10], implantable polymeric scaffolds [11,12], and neuroprotective pharmacological agents [13] for SCI has still not resulted in significant functional recovery and cure. To bridge this gap in SCI treatment, research into the use of spinal cord stimulation (SCS) to improve locomotor and/or autonomic functions following SCI has grown in prominence. Unlike neurorehabilitation strategies requiring the patient to retain some motor ability to benefit (i.e., motor incomplete patients), epidural SCS (eSCS) has also been demonstrated to restore volitional motor control in motor complete patients. The benefits of eSCS also extend beyond locomotor functional recovery to cardiovascular, respiratory, and genitourinary functions.

The mechanisms underlying eSCS for improving functional recovery in SCI have been hypothesized by computational [14,15] and preclinical studies [16,17], with therapeutic benefits reported in humans through electromyographic (EMG) studies. Clinical studies thus far have reported on different locomotor and/or autonomic outcomes in patients with different AIS grades of spinal injury and with different stimulation parameters, as adjuncts to different varying neurorehabilitation regimens, and with varying and often limited results in efficacy and safety. Nevertheless, there is tremendous momentum for technology to become more readily available to people with SCI.

To support the translation of this neuromodulation strategy into clinical practice, this review aims to summarize and discuss the current evidence on the efficacy and safety of eSCS, and the underlying mechanisms, in improving somatic motor control and autonomic function in individuals with chronic SCI. We also discuss the limitations of the current evidence and provide recommendations for future research priorities along with recommendations for the field in general.

## 2. Epidural Spinal Cord Stimulation

### 2.1. History and Evolution of eSCS

Central to the development of SCS is the “gate control theory of pain” hypothesized by Melzack and Wall in 1965. This theory postulated that stimulation of non-noxious large-diameter myelinated sensory fibers could be used to suppress noxious input from pain fibers [18]. To translate this theory into clinical practice, Wall and Sweet delivered electrical impulses to the skin and/or under the skin with a subcutaneous electrode in eight patients with different intractable pain conditions [19]. Following the improvement in these pain conditions, Shealy and Mortimer developed the first implantable SCS system, which Shealy termed “dorsal column stimulation” in 1967. This application was based on Shealy’s previous hypothesis that pain relief would be optimum by stimulating the dorsal column of the spinal cord white matter, where the proprioceptive fibers corresponding to multiple dermatomes are located [20]. As such, the dorsal column stimulator was implanted subdurally above the dorsal column of the spinal cord in adult cats and was found to provide significant pain relief through the assessment of prolonged small-fiber afferent-discharge (PSAD) stimuli.

The effects of eSCS in improving motor function were discovered serendipitously in the application of SCS to treat intractable back pain in a patient with multiple sclerosis (MS) [21]. Recovery of some volitional motor control in this patient led to subsequent studies using SCS to treat spasticity, which revealed improvements in bladder, bowel, and voluntary motor functions [22].

**Figure 1:**
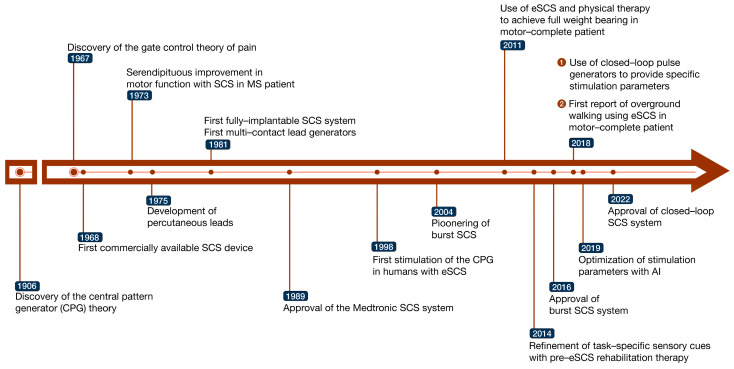
Timeline of SCS discovery and research milestones.

The application of eSCS in treating spasticity in SCI was then explored with a focus on defining the optimum stimulation site. Early studies demonstrated that placement of stimulation electrodes caudal to the lesion produced significant improvement of spasticity [23,24], which was supported by Dimitrijevic et al. in patients with cervical lesions [25]. The importance of the rostro-caudal placement of SCS electrodes relative to the lesion was subsequently emphasized in leg spasticity case studies with lumbar SCI [25,26]. The spinal circuitry target of eSCS for SCI was based on the central pattern generator (CPG) theory, which was first described by Sherrington in 1906 [27], and supported by preclinical and human studies [28,29]. Defined as neuronal circuits that produce rhythmic locomotor patterns when activated, the CPG was first stimulated by Dimitrijevic et al. in humans using eSCS to produce motor activity [30]. The CPG was stimulated in subsequent studies, which reported varying degrees of improvement in volitional motor control [31,32,33,34]. Figure 1 summarizes the timeline of SCS discovery and research milestones.

The next development in eSCS for SCI was in 2011, when Harkema et al. [22] combined eSCS with pre-implantation physical therapy in an AIS-B patient to produce independent weight-bearing standing for approximately 4 min. This study highlighted the importance of sensory cues in generating motor control. The role of sensory cues was further refined by Angeli et al. [35], who employed both task-specific auditory and sensory cues with a pre-implantation rehabilitation regimen to produce volitional motor control in AIS-A and AIS-B patients. This evidence of sensory information in improving motor activity with eSCS has since further been explored with stimulation optimization for different patients [36,37,38,39,40,41]. 

The next step in the evolution of SCS for SCI involved the method in which the stimulation was delivered. Wagner et al. [42] used a closed-loop pulse generator that produced specific stimulation parameters depending on the motor task that was being performed. This spatiotemporal eSCS system involved first identifying stimulation parameters that optimally triggered mobilization of the hip, knee, and ankle joints, and the sequential selection of these parameters to match the individual movements that produce stepping. This method of SCS delivery contrasted the earlier models based on the methodology for treating neuropathic pain, which employed continuous non-adaptive stimulation pulses to the dorsal column. The use of individualized, optimized, and adaptive stimulation parameters has since then followed recent studies in addition to different forms of neurorehabilitation to generate locomotion [43,44,45,46,47,48,49,50,51,52,53]. Notably, artificial intelligence methods for optimizing stimulation parameters have been used [44,45]. The choice of adjunct physical rehabilitation has also evolved to include the use of exoskeleton-assisted walking techniques [46,52].

Following the discovery of the importance of sensory cues in combination with eSCS by Harkema et al. [22], there has been significant progress in the techniques of eSCS. Five landmark papers have played a pivotal role in advancing our understanding on the use of eSCS in improving motor activity. Harkema et al. [22] demonstrated full weight bearing in a motor-complete patient, highlighting the crucial role of sensory cues in enhancing motor activity with eSCS. This importance was reinforced by Angeli et al. [39], who achieved overground walking in motor-complete patients, marking a milestone in the field. Gill et al. [41] emphasized the significance of task-specific training in conjunction with eSCS and were able to achieve the first independent stepping in an ASIA-A patient. The introduction of closed-loop eSCS by Wagner et al. [42] highlighted the importance of precise electrode placement. Within one week, one patient in this study regained stepping ability, showcasing the efficacy of closed-loop eSCS systems. Finally, Rowald et al. [53] were able to achieve walking within one day by using updated grids to stimulate dorsal roots. This study signified the importance of precise neural targeting and provided a rapid timeline for functional improvement.

### 2.2. eSCS Implantation Approaches

In epidural spinal cord stimulation, electrodes are implanted on the dorsum of the dura via a percutaneous or surgical approach. The percutaneous approach uses multi-contact percutaneous leads, which typically have eight individually programmable contacts. Percutaneous lead wires are directed into the epidural space under fluoroscopic guidance using the Seldinger technique with a modified Tuohy cannula [54]. The surgical approach is conducted via laminotomy using independent multi-column paddle leads, typically with 16 contacts. Both percutaneous and paddle leads can be programmed to provide combinations of mono, bi-, or multipolar stimulation. Once in the optimal anatomic position, the leads are anchored in deep tissue and connected to a subcutaneous implantable pulse generator or an externalized pulse generator [55].

### 2.3. Mechanisms of eSCS in Locomotor Control

#### 2.3.1. Spinal Networks and Reflex Pathways

The importance of the topographical organization of motor circuits of the spinal cord in locomotion has been demonstrated in eSCS studies. In addition to the supraspinal tracts, which play a role in the planning, initiation, and modulation of locomotion [56], complex interneuronal networks within the spinal cord perform important roles in locomotion through efficient signal integration and motor coordination [57,58]. The role of interneuronal networks is evident in ex vivo spinal cord preparations without supraspinal input where electrical stimulation of the spinal cord generates rhythmic flexor and extensor movement in motor nerves [59]. In SCI where motor circuits are disrupted, spared propriospinal interneurons enable communication by forming and activating circuits across the spinal cord lesion [60]. Courtine et al. [61] showed that propriospinal networks can generate functional recovery and supraspinal control of stepping in rodents with transected supraspinal tracts.

Spinal reflex pathways are also recruited by eSCS. Prominent reflex pathways include the monosynaptic (Ia, muscle spindle fibers; Ib, Golgi tendon organ fibers) and polysynaptic (II) pathways. Recruitment of Ia, Ib, and II afferents by eSCS activates motor neurons via monosynaptic and/or polysynaptic pathways [15], leading to the activation of extensors and flexors, respectively [34]. Groups I and II afferent fibers are also responsible for evoking short- and long-latency muscle responses, respectively. The generation of long-latency muscles by Group II afferent fibers have been shown to be responsible for generating stepping patterns in volitional locomotor control [62]. Animal studies also demonstrate that monosynaptic group I afferent fibers complement locomotion through enhancing postural stability and stance [63,64].

#### 2.3.2. Sensory and Proprioceptive Inputs

Sensory and proprioceptive feedback signals during movement also play a role in the mechanism of spinal cord neuromodulation. Several processes in the gait cycle are integrated with proprioceptive feedback, which activates motor neurons in the proper spatiotemporal fashion [65,66]. Electrical stimulation of proprioceptive circuits has been hypothesized to increase the excitability of spared neuronal networks and decrease their threshold potentials, preparing them for subsequent supraspinal and/or propriospinal activation. This, in essence, allows volitional locomotion to be controlled by sensory information, which requires an intense physical rehabilitation program to facilitate remodeling of the supraspinal and propriospinal pathways [16]. Sensory feedback from weight bearing has been shown to enable appropriate stepping patterns after epidural spinal cord stimulation in supraspinally transected rodents [67,68]. Lavrov et al. [69] demonstrated the laterality of sensory input from non-deafferented and deafferented models in the recovery of coordinated rhythmic hindlimb activity in rats. This led to the conclusion that eSCS facilitates stepping through ipsilateral afferents that project to locomotor networks. The group also highlighted the function of proprioceptive and cutaneous sensory inputs in promoting posture balance in rats following eSCS [70]. Takeoka et al. [71,72] have also studied the function of proprioceptive neurons using a genetic model for proprioceptive afferent ablation. The authors demonstrated that eSCS-evoked motor activity was critically dependent on muscle spindle proprioceptive afferents and proprioceptive neurons within the dorsal root ganglion.

#### 2.3.3. Central Pattern Generators (CPGs)

CPGs are spinal neuronal circuits that can generate coordinated action potentials for initiating and maintaining rhythmic activity [27]. The presence of CPGs for locomotion in animals was shown by Forssberg et al. [73] on paraplegic kittens and adult cats, who exhibited excitation of the CPGs and consequent locomotion by L-DOPA or supraspinal electrical stimulation. Coordinated stimulation of CPGs in rats has also been reported to promote adaptive plasticity in the spinal cord and spinal learning [74]. The evidence supporting CPGs has been extended to humans, where CPGs present in the lumbar spinal cord enable rhythmic activation of flexor and extensor muscles during walking following eSCS [75]. Despite the evolutionary conservation of locomotor function between different organisms, differences between rodents, larger animals, and humans should be noted in relation to the mechanism of CPGs. Several aspects of the human gait cycle incorporate complex sensory–motor behaviors, which may be the result of the spinal CPG recruiting both supraspinal and propriospinal circuits. Many studies investigating the efficacy of eSCS in locomotion have employed rehabilitation techniques that provide sensory input to presumably activate the CPG [76].

### 2.4. Mechanisms of eSCS in Autonomic Control

eSCS has shown positive effects on the cardiovascular system, including the maintenance of blood pressure and the prevention of autonomic dysreflexia. The propriospinal system can improve cardiovascular function through blood pressure stabilization with eSCS. It has been postulated that activation of dorsal root afferents by eSCS in the lumbosacral region raises the resting membrane potential of sympathetic networks, which in turn increases total peripheral resistance with a resultant rise in blood pressure [77,78]. Contrary to this theory, two groups have demonstrated and suggested that eSCS in the lumbosacral region results in a net inhibitory response, either through inhibition of dorsal neuron firing and activation or activation of inhibitory interneurons [79,80]. This net inhibitory effect provides a possible mechanism for eSCS in ameliorating autonomic dysreflexia as this physiological response arises from sympathetic activation and consequent vasoconstriction of peripheral arteries. Another hypothesized theory for the mechanism of eSCS in blood pressure stabilization relates to increased sensitivity of the baroreceptor response. eSCS may increase the stimulation of aortic arch and carotid sinus baroreceptors during orthostasis, leading to bradycardia and increased vascular tone [77,81].

The functional importance of eSCS in respiratory neurorehabilitation was discovered in animal studies following the use of eSCS to activate inspiratory and expiratory muscles in dogs [82,83] and the phrenic nerve in cats [84]. Like locomotor function, mechanisms underlying respiratory recovery with eSCS include spinal neural networks and sensory inputs. Propriospinal networks activated by eSCS play a role in appropriate respiratory muscle pattern activity through excitation and inhibition of respiratory motor neurons [85]. Reflex mono- and polysynaptic networks are also crucial components of the phrenic nerve motor system that drive respiratory motor function and the perception of breathing. Phrenic nerve afferents consist of Groups Ia, Ib, and II fibers, which are first depolarized by eSCS, exerting excitatory output to the motor circuitry and propriospinal networks to enable respiratory motor function and adaptation [15,86,87]. These Ia, Ib, and II fibers utilize afferent feedback in addition to the activated descending inputs, highlighting the importance of somatosensory input in eSCS-induced respiratory neuromodulation [85]. Additional means of activating respiratory networks include interaction of eSCS with the cerebrospinal fluid, and the recruitment and depolarization of glial cells to release glutamate and adenosine [88,89].

The topographical arrangement of neural networks in the lumbosacral region is important to understand the mechanisms of eSCS on the genitourinary system. The L1–L2 region contains sympathetic networks that function in bladder storage. Stimulation of this region activates afferent fibers, triggering a reflex pathway that inhibits bladder activity during filling [90]. Evidence from preclinical studies also highlights a control center at the L3–L4 region for the detrusor muscle and external urethral sphincter [91]. Sexual function may also be modulated at the L3–L4 regions in rodents, and L3–L5 in humans, through stimulation of neurons that mediate the ejaculation reflex [92]. Finally, stimulation of the sacral cord recruits axons from pelvic and pudendal nerves, which may stimulate or inhibit micturition [93,94]. Spinal transection studies have suggested that activation of the pudendal nerve is independent of descending inputs in improving bladder storage and voiding [95]. Additionally, sacral nerve stimulation in individuals with complete or incomplete SCI suggest the importance of spino–bulbo–spinal pathways in bladder function and continence [96]. Knowledge of these important stimulation areas has led to targeted use of stimulation parameters to elucidate the mechanism of eSCS in improving bladder storage, filling and voiding, continence, and sexual function. 

Hemodynamic instability due to SCI manifests as orthostatic hypotension and syncope, or autonomic dysreflexia with acute sustained systolic blood pressure often exceeding 300 mmHg. The lack of reflex autonomic regulation is secondary to interruption of supraspinal control of sympathetic ganglia along the thoracolumbar sympathetic chain [97]. In a recent study, Squair et al. [98] demonstrated that animal models of cervical SCI with eSCS implanted ventrally on the thoracolumbar spine not only activated an autonomic regulatory response to extreme changes in blood pressure but also activated afferent baroreceptor signaling in a synchronized neural network.

Pulmonary function in animal models of SCI improves with eSCS activation of the diaphragm and inspiratory intercostal muscles approximating spontaneous breathing [99,100,101,102]. Spontaneous breathing is characterized by asynchronous activation of respiratory muscles and a gradual recruitment of slow to fast motor units [103,104]. In contrast, to direct electric stimulation of the phrenic nerve, which results in activation of all axons and creates a synchronous contraction of diaphragm motor units, eSCS results in central control of recruited motoneuron pools and asynchronous activation of motor units. DiMarco and Kowalski [105] demonstrated that eSCS implanted in the upper thoracic region of dogs with high cervical SCI recruited inspiratory motor units with a pattern of activation under central control. The generated negative airway pressures and inspiratory capacity resulted in physiologic breathing patterns. While expiration is a passive process, expiratory muscle paresis or paralysis in patients with SCI results in an inactive mucociliary reflex. DiMarco et al. [99,100,101,102] demonstrated that eSCS leads implanted in the thoracolumbar spine (T9–L1) of patients with cervical SCI not only improved the cough reflex and clearance of bronchial secretions, but also generated expiratory flow rates and airway pressures that improved inspiratory and total lung capacity.

## 3. Methods

### 3.1. Search Strategy

This review was reported according to the PRISMA guidelines [106]. A comprehensive search of published literature was conducted in MEDLINE/PubMed, Embase, Web of Science, and IEEE Xplore electronic databases in September 2023 by two reviewers (EM and JC). The searches were conducted independently and blindly to ensure reliability. The systematic search included the following terms: (“spinal cord injury” OR “SCI”) AND (“spinal cord stimulation” OR “SCS” OR “eSCS” OR “epidural stimulation” OR “epidural electrical stimulation” OR “EES”) as both keywords and Medical Subject Headings (MeSH) terms. Additionally, references of studies meeting the inclusion criteria were manually reviewed to supplement the results of the electronic searches.

### 3.2. Study Selection Criteria

Selection eligibility was governed by the PICOS (Population, Intervention, Comparison, Outcome, and Study) design, which yielded the following inclusion criteria: (1) human subjects older than 18 years of age with a diagnosis of SCI; (2) the use of a surgically implanted epidural spinal cord stimulator; (3) assessment of motor and/or autonomic responses; and (4) study design was a case series, case study, cohort study, or randomized control trial (RCT). Exclusion criteria included: (1) animal studies; (2) alternative types of electrical spinal cord stimulation; (3) studies primarily assessing chronic pain, spasticity or other outcomes; (4) studies with insufficient data to be extracted, including reviews, abstracts, editorials, and proposed research protocol descriptions; and (5) non-English articles.

### 3.3. Data Extraction

Two independent reviewers (EM and JC) screened both abstracts and titles and full texts of results yielded by the search strategy. Standardized data were extracted from eligible studies according to the following metrics: first author’s name and year of publication, patient demographics (age and gender), clinical characteristics (level of injury, time since injury, and ASIA classification), stimulator characteristics (type of device/manufacturer, number of leads, location of leads, and method of lead placement), stimulation parameters (frequency, pulse width, amplitude, stimulation time length, and optimization), whether the subjects enrolled in a rehabilitation program, assessed locomotion and/or autonomic outcomes, and adverse effects.

### 3.4. Analysis of Locomotor Outcomes

Studies reporting locomotor outcomes were reviewed in detail to identify non-overlapping cases/cohorts. Overlapping cases/cohorts were identified by comparing any published subject ID number across publications or self-reference to a prior article with published case(s). When multiple articles reported on the same case/cohort, success rate was extracted from the most recent article. Articles with overlapping cases/cohorts were excluded. Studies reporting only EMG outcomes were removed. From the remaining studies, a success rate of eSCS in achieving locomotor outcomes was calculated for each article and a cumulative success rate was calculated across all included studies. An effect size was not calculated due to the small sample size and lack of reporting of consistent quantitative outcomes with confidence intervals for locomotor outcomes in individual studies.

### 3.5. Bias Assessment

Two independent reviewers, E.M. and J.C., assessed bias using the Risk Of Bias In Non-Randomized Studies of Interventions (ROBINS-1) tool by the Cochrane Scientific Committee for non-randomized studies of effects of interventions. Results of this assessment are detailed in Supplementary Table S1.

## 4. Results

### 4.1. Study Selection

**Figure 2:**
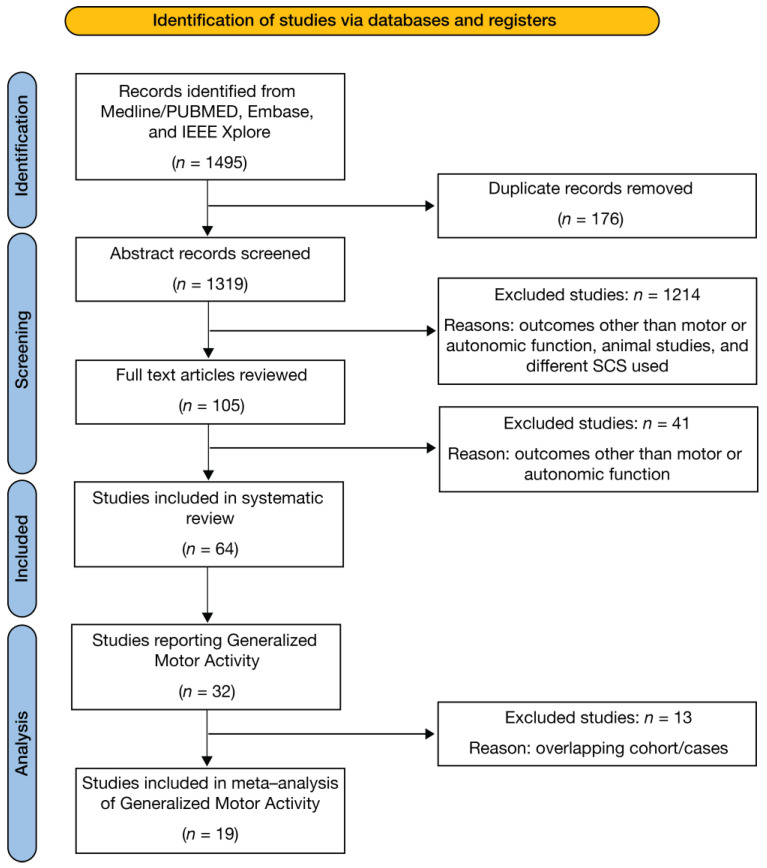
PRISMA flow diagram describing the selection process.

The initial search strategy yielded 1495 relevant articles. A total of 176 duplicates were excluded, following which 1255 articles were excluded after title, abstract, and full-text review (due to exclusion criteria). Finally, 64 studies were included in the review. Figure 2 shows the flowchart of study selection for this review.

### 4.2. Study and Participant Characteristics

Characteristics of the studies and participants are detailed in Table 1. Included studies varied by geographical location, with 46 from the USA, 7 from Austria, 5 from Canada, 3 from Switzerland, 2 from India, and 1 from Russia. Publication year of the included studies ranged from 1986 [107] to 2023 [108,109,110,111,112,113], with patient size ranging from 1 to 33. 

The total number of participants in the 64 included studies was 306, although it should be noted that some participants were subjects in more than one study, as suggested by authors and patient and clinical characteristics. Sixty-one studies adequately reported the sex of their participants: 249 were male and 49 were female. Patient age ranged from 18 to 66 years old. The shortest time since SCI was just 15 days [114] and the longest was 37 years [102]. The injury levels of included participants were mainly in the cervical and thoracic regions, with the highest injury level being C2 [102,108,114,115] and the lowest being L1 [116]. Of studies that adequately reported the AIS of participants, the majority were AIS-A (*n* = 168), followed by AIS-B (*n* = 72), AIS-C (*n* = 16), and AIS-D (*n* = 2); notably, Monshonkina et al. [116] reported two participants with AIS-A/B and 1 with AIS-B/C.

### 4.3. Stimulator Parameters, Optimization, and Physical Rehabilitation

Treatment characteristics are summarized in Table 2. There was great variation between stimulation device, placement, and optimization settings. The most common stimulator device was the Medtronic^®^ stimulator, which featured in 51 studies; other devices included Abbott (*n* = 3), Ardiem Medical (*n* = 1), Boston Scientific (*n* = 1), Clinical Technology Corporation (*n* = 1), Cooner Wire Co. (*n* = 1), and Neuro–Control Corp (*n* = 2). Electrodes applied ranged from 1–16 and were either paddle (*n* = 41), percutaneous (*n* = 21), or unspecified (*n* = 2) leads. The highest level of lead placement was in the C4/C5 region [117] and the lowest at S2 [39,45,46,47,53,62,116]. The most common location for lead placement was at the T11–L1 level. Lead placement location showed some consistency in terms of outcomes produced, with efficacy reported at the T9–T11 for pulmonary functions, T11–L1 for volitional motor control and cardiovascular functions, and L1–S1 for genitourinary functions. Frequencies of 0.2–400 Hz, pulse widths of 150–1000 µsec, and amplitudes of 0.1–40 V/0.1–15 mA were used across the 64 included studies. Optimization settings for stimulation also varied greatly; strategies included optimizing for paresthesia and spasticity in the earliest studies, testing a wide range of parameters and selecting the best responses, optimizing at an individual level with continuous appraisal of stimulation parameters, spatiotemporal optimization using rehabilitation training, and the use of machine learning methods.

Physical rehabilitation therapy was described in 29 studies, with 16 studies enrolling participants in both pre- and post-eSCS neurorehabilitation training, 4 enrolling in only pre-implantation rehabilitation, and 9 enrolling participants in post-implantation rehabilitation therapy only.

### 4.4. Outcomes Measured

Studies reporting locomotor function following eSCS are summarized in Table 3. Specific motor assessments included assisted/independent standing or stepping (A/I), body weight support (BWS), electromyography (EMG), gait analysis (GA), general muscle activity (GMA), increased walking speed (IWS), overground walking (OGW), proprioception, sense of effort, spasticity, sit-to-stand movements (STS), and treadmill stepping/walking (TSW). All studies assessed lower limb motor control with the exception of Lu et al. [117], which evaluated volitional hand motor function (grip and control) in tetraplegic individuals. EMG (*n* = 38) and GMA (*n* = 32) were the most commonly used locomotor assessment modalities. Most studies reported an overall improvement in assisted/independent locomotion in all participants, including standing, stepping, body weight support, and cycling. Six studies re-assessed the ASIA grade post intervention, with improved scores seen in four studies: Angeli et al. [39] and Lu et al. [117] both reported a reclassification from AIS-B to AIS-C in one participant; Wagner et al. [42] reported a reclassification from AIS-C to AIS-D; and Kandhari et al. [114] reported a reclassification from A-C in 8 patients, and A-D in two patients. Four studies [39,42,114,117] reported neurologic assessment of motor strength of individual muscle groups with Medical Research Council (MRC) 6-point scale pre- and post-eSCS as part of the motor section of AIS.

Nineteen articles with non-overlapping cases/cohorts were identified with a total of 78 patients. Table 4 summarizes the outcomes of these non-overlapping cohorts for A/I, BWS, GMA, IWS, and OGW. Successful self-assisted or independent stepping or standing was reported in 44% of patients. Four articles [33,42,53,118] reported decreased BWS from 100% to a range of 0–60% during stepping/walking after eSCS implantation. The average success rate of GMA with eSCS turned on was 87%. Notably, the average success rate of GMA was markedly decreased by two studies, Barolat et al. [107] and Smith et al. [115], who reported success rates of 14.3% and 9.1%, respectively; excluding these studies, the success rate of GMA was 96% in the remainder 14 studies. Increased walking speed was reported in 65% of patients, and OGW was improved in 80% of patients who could be assessed for OGW. OGW was examined in four studies, and in these studies, 10 patients were able to achieve a stage at which OGW could be assessed, of which 8 improved [33,42,53,109].

Table 5 summarizes the autonomic function outcomes following eSCS. Autonomic outcomes according to body system were cardiovascular (blood pressure, orthostasis, heart rate, cardiac function, and plethysmography), pulmonary (airway pressures, peak expiratory flow rates, spirometry, and volume of respiratory secretions), and gastrointestinal and genitourinary (bowel function, bladder incontinence, bladder storage and voiding, sexual function, and urodynamics). A total of 12 studies assessed cardiovascular function, 6 pulmonary function, and 7 genitourinary function following eSCS. All studies assessing autonomic function with eSCS reported improvement in outcomes except the following: Katz et al. [119] reported an insignificant change in urodynamic parameters in 17 patients; Beck et al. [120] reported worsening of bladder continence with eSCS optimized for motor function; Herrity et al. [121] reported elevated BP during bladder distension. Additional outcomes included improved middle cerebral artery (MCA) blood flow [78], body composition [120,122], sexual function and orgasm [22,45], and quality of life [108,123].

### 4.5. Methodological Quality

Most studies included in this review (53%) were case reports or small case series: 18 reports of a single case each, 11 case studies with two patients, and 5 case studies with three patients. Only 14% of the included articles had a sample size greater than or equal to 10 participants. One of the published studies was reported as a consecutive case series [112], while none were case–control, cohort, or randomized controlled studies. Accordingly, all studies reported in the literature currently provide Level 4 evidence [124].

Although several of the studies reported results from trials registered on Clinicaltrials.gov [39,40,41,43,45,102,109], most of the currently published data were from early feasibility analyses or well-selected cases. Multiple studies reported outcomes with eSCS turned ON and eSCS turned OFF [42,47,53,78,98,113,125] and patients serving as their own controls of pre- and post-eSCS implantation. Only a few studies mentioned that there were other SCI patients who were tested with eSCS without a positive effect but did not describe the characteristics or outcomes of excluded patients, creating a potential publication bias [107].

Finally, most articles utilized qualitative assessments as the primary outcomes (Table 3). Only four articles reported individual muscle group strength of a pre- and post-eSCS on the AIS [39,42,114,117]. Nevertheless, most studies included EMG as an objective assessment of muscle conductivity in addition to reported biomechanics and subjective assessment of function.

Using the ROBINS-I tool (Supplementary Table S1), the three pre-intervention domains (bias due to confounding, selection of patients, and classification of interventions) ranged from moderate to serious. Of these, a judgement of serious bias was seen in 16/19 studies for confounding, and 9/19 for both selection of patients and classification of interventions. For post-intervention domains, most studies had a low risk of bias in deviations from intended interventions (15/19), bias in due to missing data (18/19), and bias in selection of reported result (12/19). Notably, all studies (19/19) scored a serious risk of bias in measurement of outcomes, primarily due to a lack of blinding reporting by studies, as well as the difficulty of blinding with spinal cord stimulation.

### 4.6. Adverse Effects

In addition to risks associated with surgery, commonly expected adverse events of eSCS include paddle migration [126], infection, seroma, and hematoma [127,128]. Seven articles reported 24 adverse events (Table 3 and Table 5) including a lead migration [52]; a hip fracture during training [39]; a metatarsal fracture and a pressure ulcer [47]; a wound drainage, one case of cellulitis, a device infection, two seromas, two wound dehiscence, two cases of ileus, and two wound infections [108]; two cases of post-implant autonomic dysreflexia [123]; three wound washouts [108]; and discomfort/pain in three patients [31,36,39]. There were ten reported 30-day post-surgical complications [108,123]. Patients with wound/device infections, wound washout, device migration, or fractures required inpatient medical or surgical intervention to manage serious adverse events. 

## 5. Discussion

### 5.1. Efficacy of eSCS in Improving Locomotor Function

Published studies investigating the effect of eSCS on locomotor function have demonstrated high success rates in patients with SCI. The average success rate of eSCS across studies for GMA was 89% (CI: 0.924, 0.9331). All studies with BWS outcomes reported decreased BWS, and two reported zero BWS required for independent stepping/standing when eSCS was turned on. Most patients for whom eSCS stimulation was reported as successful were able to perform independent stepping/standing with decreased BWS. Some studies reported functional improvement outside of the lab including community ambulation and improved ability to perform tasks at home. Most patients however, required significant external assistance with motor function from physical therapists. This ranged from joint stabilization to pelvis stabilization to manually imposing stepping movements [118]. Nine studies reported that OGW forces were increased with eSCS turned on, but recorded forces were sometimes asymmetric with bilateral array turned on and vice versa. In addition, EMG measures at muscle extremities was consistently increased after eSCS across studies.

An overwhelming majority of studies did not report individual muscle strength measures on the well-validated, highly reliable AIS. Two of the studies that included AIS as an outcome reported patients with muscle strength of zero among patients who were noted to otherwise stand/step or walk with decreased/minimal support. This may be explained secondary to the balanced contraction of agonist and antagonist muscles by suprathreshold motor action potential generated with eSCS.

### 5.2. Efficacy of eSCS in Improving Autonomic Function

The majority of the articles included in this review present evidence for the efficacy of eSCS in regulating autonomic dysfunction secondary to SCI. Although earlier studies demonstrated variable effects of eSCS and several device failures, the technology and surgical practice have since developed. Katz et al. [119] reported no changes in urodynamics after eSCS in most of their SCI patients. However, later research indeed demonstrates that eSCS improves bladder [22,45,121] function and results in reduced residual post-void volume and more efficient [129] reflexive voiding capacity. An important caveat to eSCS for bladder function should, however, be considered. Specific stimulation parameters for bladder function differ from the parameters required for motor control. Beck et al. [120] demonstrated that bladder function in their patients was worsened when the stimulation parameters were optimized for motor control. In contrast, stimulation parameters for motor control appear to improve cardiovascular function [130], and this appears to be mediated via activation of a sympathetic response [98]. In fact, multiple articles demonstrate the efficacy of eSCS for blood pressure and heart rate control in both patients with orthostatic hypotension [78,109,130,131] and autonomic dysreflexia [113,121]. Finally, several articles [99,100,102,114,123] reported the efficacy of eSCS on respiratory function, including improving cough reflex, recruitment of intercostal muscles, airflow dynamics, and pulmonary function parameters. Although the majority of the articles included in this review demonstrate positive effects of eSCS on autonomic function, these results should be considered with a caveat, as the majority of articles on any specific autonomic function are published from the same research group. Nevertheless, with the proper stimulation parameters and localization of the epidural electrodes, eSCS appears to be effective in improving autonomic function in patients with SCI.

### 5.3. Efficacy of eSCS in Improving Combined Locomotor and Autonomic Function: AIS Scores

Pre-intervention AIS scores were mostly AIS-A (168/306), which indicated a complete lack of motor and sensory function below the level of injury. The impact of eSCS on the improvement of voluntary motor, and possibly sensory, functions are highlighted by reclassifications from AIS-A to AIS-C in 10 patients [39,114,117] and AIS-A to AIS-D in 2 patients [114], highlighting that eSCS can partially restore neurological functions lost due to SCI. Notably, the transition from complete to incomplete SCI status (e.g., from AIS-A/B to AIS-C/D) represents a significant improvement in quality of life, as it may correlate with regained voluntary control over motor functions, enhanced sensory perception, and reduced dependence on assistive technologies or caregivers. Given the limited treatment options available for SCI that offer substantial improvements in motor and sensory functions, the reclassification in AIS status seen with some eSCS patients is significant. The observed variability in eSCS outcomes, as evidenced by the range of AIS score improvements, emphasizes the need for personalized treatment approaches. Understanding the factors that influence recovery, such as intervention timing post-injury, baseline neurological status, and specific stimulation parameters, will be crucial for optimizing eSCS protocols.

### 5.4. Safety of eSCS in SCI

The device safety of eSCS has been optimized over the past two decades as an effective FDA-approved interventional pain management tool. The International Neuromodulation Society has published a consensus guidance on the safety, risks, and steps to reduce complications related to eSCS and note that eSCS is a generally safe, minimally invasive procedure [128]. The most reported device-related complications in this review include expected device complications such as paddle migration, device infection, and perioperative surgical complications (seroma, wound infection, wound dehiscence) [128]. There are, however, some potential differences in the safety profile of eSCS between patients with severe pain who are fully ambulatory and patient with upper/lower extremity paralysis. Patients with paralysis attempting to stand or take steps with decreased external support are at a risk of falls, fractures, hematoma, and hospitalization arising thereof. Moreover, patients with paralysis secondary to SCI are at a higher risk of osteopenia and osteoporosis [132] and may be prone to fragility fractures [133] with initial attempts at unassisted standing/stepping. Although eSCS devices have built-in safety features, stimulation settings and location of the implanted paddle for motor/autonomic function are, at present, not established, presenting a potential risk of nerve/muscle damage and treatment failure.

**Table 1:** Summary of patient characteristics.

Author (Year)		Country	Subjects (Sex)	Age Range	Injury Level	Years Since Injury	ASIA Score
**1**	Barolat (1986) [107]	USA	1 (M)	22	C5	0.75	C
**2**	Katz (1991) [119]	USA	33 (31M, 2F)	24–66	C4–C10	0.58–31.5	A–D
**3**	Dimitrijevic (1998) [30]	Austria	6 (3M, 3F)	18–58	C5–T8	1–5	A
**4**	Herman (2002) [32]	USA	1 (M)	43	C5–C6	3.5	C
**5**	Cahart (2004) [31]	USA	1 (M)	43	C5–C6	3.5	C
**6**	Jilge (2004) [134]	Austria	5 (2M, 3F)	24–34	C4–T10	2–8	4A, 1B
**7**	Minassian (2004) [34]	Austria	10 (7M, 3F)	18–58	C4–T10	2–8	8A, 2B
**8**	Ganley (2005) [135]	USA	2 (M)	43–48	C6–T8	3.5–8	C
**9**	DiMarco (2006) [99]	USA	1 (M)	52	C5–C6	7	C
**10**	Huang (2006) [33]	USA	2 (M)	43–48	C5–T8	3.5–8	C
**11**	Minassian (2007) [136]	Austria	15	**–**	**–**	**–**	A
**12**	DiMarco (2009) [100]	USA	9 (8M, 1F)	23–52	C3–C6	1–34	–
**13**	Harkema (2011) [22]	USA	1 (M)	23	C7–T1	3.4	B
**14**	Monshonkina (2012) [116]	Russia	4 (1M, 3F)	22–58	C5–L1	–	2 A/B, 1B, 1 B/C
**15**	Minassian (2013) [118]	Austria	7	–	–	–	–
**16**	Angeli (2014) [35]	USA	4 (M)	23–32	C6–T6	2.2–4.2	2A, 2B
**17**	Sayenko (2014) [62]	USA	3 (M)	23–32	C7–T4	2.2–4.2	1A, 2B
**18**	Danner (2015) [137]	Austria	10 (7M, 3F)	18–58	C4–T10	2–8	6A, 4B
**19**	Hoefstoetter (2015) [138]	Austria	8 (6M, 2F)	18–33	C5–T6	1–13	6A, 2B
**20**	Rejc (2015) [36]	USA	4 (M)	24–33	C7–T4	2.2–4.2	2A, 2B
**21**	Lu (2016) [117]	USA	2 (M)	18–20	C5–C6	2–2.5	B
**22**	Grahn (2017) [139]	USA	1 (M)	26	T6	3	A
**23**	Rejc (2017) A [37]	USA	1 (M)	32	C7	4.2	B
**24**	Rejc (2017) B [38]	USA	4 (M)	24–33	C7–T4	2.2–4.2	2A, 2B
**25**	Angeli (2018) [39]	USA	4 (3M, 1F)	22–32	C5–T4	2.5–3.3	2A, 2B
**26**	Aslan (2018) [140]	USA	7 (M)	–	C5–T4	2.0–3.5	4A, 3B
**27**	DiMarco (2018) [101]	USA	1 (M)	50	C4	2	–
**28**	Formento (2018) [40]	Switzerland	3 (M)	28–47	C4–C7	4–6	2C, 1D
**29**	Gill (2018) [41]	USA	1 (M)	26	T6	3	A
**30**	Harkema (2018) A [78]	USA	4 (3M, 1F)	24–35	C4	3.8–8	3A, 1B
**31**	Harkema (2018) B [131]	USA	4 (3M, 1F)	24–35	C4	3.8–8	3A, 1B
**32**	Herrity (2018) [129]	USA	1 (M)	(31)	C5	–3.3	B
**33**	Wagner (2018) [42]	Switzerland	3 (M)	28–47	C4–C8	4–6	2C, 1D
**34**	Walter (2018) [125]	Canada	1 (M)	32	C5	4	B
**35**	West (2018) [78]	Canada	1 (M)	Early 30s	C5	–	B
**36**	Calvert (2019) [43]	USA	2 (M)	26–37	T3–T6	3–6	A

**Table 2:** eSCS treatment characteristics.

Author (Year)		Stimulator Type	Lead Placement	No. of Leads	Lead Levels	Stimulation Parameters			Stimulation Optimization	Rehabilitation	
						Frequency	Pulse Width (μs)	Amplitude		Pre–Op	Post–Op
**1**	Barolat (1986) [107]	Clinical Technology Corporation	PC	1	T1–T2	75 Hz	250	–	Stimulation parameters optimised for paraesthesia. Frequencies of 30–100 Hz were tested	No	No
**2**	Katz (1991) [119]	Medtronic	Paddle	4	–	–	–	–	Parameters optimized for spasticity	No	No
**3**	Dimitrijevic (1998) [30]	Medtronic	–		T11–L1	25–50 Hz	200–500	5–9 V	Muscle twitches were tested using 3 cathode leads, followed by testing frequencies of 1–120 Hz and amplitude 1–10 V	No	No
**4**	Herman (2002) [32]	Medtronic	PC	4	LS enlarge–ment	–	–	–	A variety of electrical parameters were tested for efficacy in promoting gait		
**5**	Cahart (2004) [31]	Medtronic	PC	4	T10–T12	40–60 Hz	800	Midpoint between sensory and motor thresholds	A wide range of parameters were tested, with selection of pulse widths > 500 μs and frequencies of 40–60 Hz	Yes	Yes
**6**	Jilge (2004) [134]	Medtronic	PC	4	T12–L1	5–60 Hz	210–450	1–10 V	Muscle twitches were elicited using a single electrode, with the stimulation amplitude being increased to the point of eliciting brief muscle contractions	No	No
**7**	Minassian (2004) [34]	Medtronic	PC	4	T10–L1	2.2–50 Hz	–	1–10 V	Optimized for spasticity by applying strengths of 1–10 V at frequencies 2.2–100 Hz using different contact combinations of electrodes	No	No
**8**	Ganley (2005) [135]	–	PC	4	T10–T12	20–60 Hz	800	Between sensory and motor thresholds in S1 and at motor threshold in S2	Parameters adjusted on an individual basis	Yes	No
9	DiMarco (2006) [109]	Neuro–Control Corp	PC	1	T9, T11, L1	53 Hz	150 –200 μs	40 V	Pulse width of 150 μs at T9, and 200 μs at T11 and L1.	No	No
10	Huang (2006) [33]	Medtronic	PC	4	T10–L2	20–40 Hz	800	3–8.5 V	Stimulation intensity was set between sensory threshold and motor threshold but closer to motor threshold, during gait training sessions		
11	Minassian (2007) [136]	Medtronic	PC	4	T10–L1	2.2–50 Hz	210	1–10 V	–	No	No
12	DiMarco (2009) [100]	Neuro–Control Corp	PC	1	T9, T11, L1	30–40 Hz	150–200	30–40 V	–	No	No
13	Harkema (2011) [22]	Medtronic	Paddle	16	L1–S1	5–40 Hz	210 or 450	0.5–10 V	Variable combinations were tested to optimize standing and stepping. 15 Hz, 8V of the caudal level (L5–S1) was used for standing caudal; 30–40 Hz and sensory cues for manually facilitated stepping	Yes	Yes
14	Monshonkina (2012) [116]	Cooner Wire Co.	PC	2–4	L2–L4, S2	1–12 Hz	**–**	**–**	Therapeutic mono/bipolar (stimulation frequency of 1–12 Hz) 2 times for 30 min in addition to routine pharmacotherapy	Yes	No
15	Minassian (2013) [118]	Medtronic	PC	4	Lumbar spinal cord	2–42 Hz	**–**	**–**	–	No	No
16	Angeli (2014) [35]	Medtronic	Paddle	16	L1–S1	25–30	**–**	**–**	Stimulation parameters optimized for each leg and joint movement, with optimal frequency set at either 25 or 30 Hz	Yes	Yes
17	Sayenko (2014) [62]	Medtronic	Paddle	16	L1–S2	2 Hz	210	0.5–10 V	Bilateral-evoked potentials from leg muscles were collected and evaluated by spatial, temporal, and amplitude characteristics to optimize location and symmetry of electrode placement	No	No
**18**	Danner (2015) [137]	Medtronic	PC	4	T11–L1	2–130 Hz	210	0–10.5 V	–	No	No
**19**	Hoefstoetter (2015) [138]	Medtronic	PC	4	T11–L1	2–130 Hz	210	0–10.5 V	–	No	No
**20**	Rejc (2015) [36]	Medtronic	Paddle	16	L1–S1	25–60 Hz	–	1.0–9.0 V	For standing, a sub–motor threshold of 25 Hz was used followed by adjustments to activate specific motor neuron pools	Yes	Yes
**21**	Lu (2016) [117]	Boston Scientific	Paddle	16	C4/C5–T1	2–40 Hz	210	0.1–10.0 mA	Different bipolar electrode configurations were tested to identify electrode pairs with greatest hand motor responses	No	Yes
**22**	Grahn (2017) [139]	Medtronic	Paddle	16	Lumbar enlarge–ment	15–40 Hz	210	0–6	Tested wide–field vs. local–field electrode configurations using a pre–selected algorithm. Frequencies used were 25 and 40 Hz (for volitional control and stepping) and 15 Hz (for standing)	Yes	Yes
**23**	Rejc (2017) A [37]	Medtronic	Paddle	16	L1–S1	30–65 Hz	–	0.4–3.5 V	The following electrode configurations were used for the following activities: (1) standing: combination of 40–60 Hz and 0.6–1V at T1–T2 and T3–T8; (2) stepping: 30–55 Hz and 0.7–3.5 V at T2–T3, T5–T6 and T7–T9; and (3) voluntary movement: 30–65 Hz and 0.4–2.2 V at T1–T3	Yes	Yes
**24**	Rejc (2017) B [38]	Medtronic	Paddle	16	L1–S1	15–60 Hz	–	1.2–10 V	Parameters were optimized to generate continuous EMG patterns for standing after stand training.	Yes	Yes
**25**	Angeli (2018) [39]	Medtronic	Paddle	16	L1–S1/S2	5–50	–	1–10 V	Simulation parameters were optimized for each individual to achieve the best motor performance task. Both standing and stepping configurations were modified every 2–4 weeks	Yes	Yes
**26**	Aslan (2018) [140]	Medtronic	Paddle	16	T11–L1	2–35	–	0–10 V	Unique electrode configurations for each subject were used. For EMG and cardiovascular response to rostral and caudal configuration of the electrode, a constant frequency of 2 Hz was used while amplitude increased from 0–10 V	No	No
**27**	DiMarco (2018) [101]	–	PC	2	T9–T11	50	200	40 V	–	No	No
**28**	Formento (2018) [40]	Medtronic	Paddle	16	Lumbo–sacral	40	–	3–9 mA	Different frequencies and amplitudes were tested in random order to characterise the ability of eSCS to modulate motor output	No	No
**29**	Gill (2018) [41]	Medtronic	Paddle	16	T11–L1	20–25	210	3.3–6 V	Initial frequency based on prior literature. Subsequently, parameters and configurations were modified to enable voluntary control	Yes	Yes
**30**	Harkema (2018) A [77]	Medtronic	Paddle	16	T11–L1	–	450	–	Configurations were optimized to maintain a target SBP of 110–120 mmHg or 105–115 mmHg and then adjusted as needed	No	No
**31**	Harkema (2018) B [131]	Medtronic	Paddle	16	T11–L1	–	450	–	Configurations were optimized to maintain a target SBP of 110–120 mmHg or 105–115 mmHg and then adjusted as needed	No	No
**32**	Herrity (2018) [129]	Medtronic	Paddle	16	L1–S1	5–30	450	Increased in steps of 0.1 V	Stimulation parameters were initiated using a global configuration that satisfied 4 rules, including the use of a fixed frequency (from 5 Hz) and pulse width (450 μs), with voltage ramped up slowly (0.1 V increments	Yes	Yes
**33**	Wagner (2018) [42]	Medtronic	Paddle	16	T11–L1	20–129	–	0.6–8 mA	Configurations were tested as monopolar pulses in EMG with selected configurations further tested for joint torque production.	No	Yes
**34**	Walter (2018) [125]	Medtronic	Paddle	16	T11–L1	25–45	300–450	4–7 V	The frequency and pulse width were pre–set, but the participant can use the stimulator as needed	No	No
**35**	West (2018) [78]	Medtronic	Paddle	16	T11–L1	35	300	3.5 V	A series of tests was conducted over 2 weeks to determine optimum stimulation parameters to increase blood pressure	No	No
**36**	Calvert (2019) [43]	Medtronic	Paddle	16	T11–L1	40	210	0–10 V	Electrode configurations from previous literature were used to assess volitional activity	Yes	No
**37**	Cheng (2019) [44]	Medtronic	Paddle	16	L1–S1	25	200	–	The choice of stimulating electrodes was modified using a machine learning algorithm to search for optimal stimulation patterns	Yes	No
**38**	Darrow (2019) [45]	Abbott	Paddle	16	L2–S2	16–400	200–500	2–15 mA	Tested eSCS settings at each visit were chosen as the best by the participant’s experience over each month from an objectively determined setting list provided by a Bayesian optimization	No	No
**39**	Nightingale (2019) [130]	Medtronic	Paddle	16	T11–L1	35–40	300–420	3.5–6.0 V	Abdominal settings: 40 Hz, 420 μs, 3.5–6.0 V; Cardiorespiratory settings: 35 Hz, 300 μs, 3.5–6.0 V;	No	No
**40**	Terson de Paleville (2019) [122]	Medtronic	Paddle	16	L1–S1	10–45	–	–	Stimulation configurations were specific for each individual, with specific configurations selected to enable the specific motor task. Standing configuration ranged from 10–40 Hz, and stepping 25–45 Hz	Yes	Yes
**41**	DiMarco (2020) [102]	–	PC	2	T9–T11	50	200	30–40 V	Stimulus parameters were set based on previous studies, which resulted in near maximal positive airway pressure generation	No	No
**42**	Gorgey (2020) [46]	Medtronic	Paddle	16	T12–S2	40	420	6–7 V	Parameters were modified based on patient performance	No	Yes
**43**	Penã Pino (2020) [47]	Abbott	Paddle	16	L2–S2	–	–	–	Participants were provided with a programmer and allowed to adjust specific stimulation settings for specific tasks such as volitional movements, spasticity control, core strength, and autonomic functions	No	No
**44**	Beck (2021) [120]	Medtronic	Paddle	16	T12–L1	–	–	–	An optimization period of 3 weeks was used to determine task–specific parameters, which were adjusted throughout a 12–month period	Yes	Yes
**45**	Calvert (2021) [48]	Medtronic	Paddle	16	T11–L1	0.2–2	210	0–10 V	Electrode configurations were defined empirically based on the motor output of each patient that enabled specific motor activation	Yes	Yes
**46**	DiMarco (2021) [123]	Ardiem Medical	PC	2	T9–T11	50	200	20–30 V	Each subject self–selected the number of stimulations and voltages applied. Typically, 2–3 applications of SCS (20–30 V, 50 Hz, 0.2 pulse width) were applied every 2–7 min and repeated several times	No	No
**47**	Gill (2021) [141]	Medtronic	Paddle	16		20–30	200–450	2.0–4.1 V	Stimulation parameters were adjusted incrementally during initial sessions of stimulation–enabled task–specific training, and refined during BWST training sessions	Yes	Yes
**48**	Herrity (2021) [121]	Medtronic	Paddle	16	L1–S1	–	–	–	–	No	Yes
**49**	Ibánēz (2021) [49]	Medtronic	Paddle	16	T11–L1	10–40	450–1000	1.8–8.6 mA; 8 V	Parameters were optimized based on individualized maps of motor pools activation, previous evidence of lower limb extension pattern generation, and topographical organization of the activation pattern	No	No
**50**	Linde (2021) [50]	Medtronic	Paddle	16	Lumbo-sacral	–	–	–	Stimulation parameters optimized for movement were determined by participants	Yes	Yes
**51**	Mesbah (2021) [51]	Medtronic	Paddle	16	T12–L2	2 or 30	450 or 1000	Increased from 0.1 V–0.5 V with 0.1 V	Stimulation parameters were further optimized for individual joint movement	No	No
**52**	Squair (2021) [98]	Medtronic	Paddle	16	T10–L1	120	450	0–7.5 mV	Parameters were optimized to recruit lower spinal segments and to increased blood pressure	No	No
**53**	Gorgey (2022) [52]	Medtronic	PC	8	T11–T12	2–40	150–210	0–10 V	Stimulation parameters were initially set at 2 Hz, 150 μs and 0–10 V. They were subsequently optimized to 20–30 Hz and 210 μs to ensure target achievements of functional movements in the supine position	No	No
**54**	Herrity (2022) [142]	Medtronic	Paddle	16	L1–S1	15–90	300–1000	0–12 mA	Bladder storage and voiding parameters were optimization tested and refined to build cohorts for multisystem stimulation. Parameters were: (1) bladder compliance: 60 Hz, 0–5 mA, 450 μs; and (2) voiding: 30 Hz, 4 mA, 1000 μs	No	No
**55**	Kandhari (2022) A [114]	Medtronic	PC	8	T1–T5	40	210	0–3.5 V	Different stimulation settings were tested over a period of 2 weeks	No	No
**56**	Kandhari (2022) B [143]	Medtronic	Paddle	16	T11–L1	15–60	210–400	1–6 V	A self–training program was implemented with sub–threshold stimulation levels at 60 Hz, 1–1.5 V and 270 μs to maintain the excitability of spinal neural networks	Yes	Yes
**57**	Rowald (2022) [53]	Medtronic	Paddle	16	T12–S2	20 or 100	500	0.5 V	Stimulation parameters were optimized based on responses elicited by eSCS, which where then optimized for each participant. These parameters were further fine–tuned through a stimulation scheduler software.	No	Yes
**58**	Smith (2022) [115]	Medtronic	Paddle	16	Lumbo-sacral	–	–	–	Individualized maps of motor pools activation were generated followed by selection of stimulation parameters based on guidelines	No	No
**59**	Boakye (2023) [108]	Medtronic	Paddle	16	T11–L1	2	450	Increased from 0.1–0.5 V with 0.1 V	Initial testing of rostral and caudal electrode configurations was done to assess activation sequence of lower extremity muscles. Re–testing of these configurations allowed optimization of rostral muscles	No	Yes
**60**	Gorgey (2023) A [109]	Medtronic	PC	2–8	T10–L2	2–40	250–1000	1–10 mA	Spinal mapping was done daily after temporary (1 week) and permanent (2 weeks) implantation, as well after the first 6 months of the study (4 weeks) to identify optimal configurations to enable multiple functions and movements without inducing unwanted activity	No	Yes
**61**	Gorgey (2023) B [110]	Medtronic	PC	2–8	Lumbo-sacral enlarge-ment	2–25	250–1000	1–10 mA	Configurations were tested at 2 Hz at three pulse widths (250 μs, 500 μs and 1,000 μs) at current 1–10 mA. For exoskeleton–assisted walking, configuration was optimized at 25 Hz, 250 μs, and 3 mA	No	Yes
**62**	Gupta (2023) [111]	–	–	16	Lumbo-sacral	14–90	210–350	–	–	No	No
**63**	Hoover (2023) [112]	Abbott	Paddle	–	–	–	–	–	–	No	Yes
**64**	Samijema (2023) [113]	Medtronic	Paddle	16	Lumbo-sacral	17–35	300–500	4–6.8 V	–	No	No

LS: lumbosacral; PC: percutaneous; SBP: systolic blood pressure. PC: percutaneous; SBP: systolic blood pressure.

**Table 3:** Assessment and outcomes of locomotor function following eSCS.

Author (Year)		A/I	BWS	EMG	GA	GMA	IWS	OGW	Prop	SoE	Spas	STS	TSW	Key Findings	Complications
**1**	Barolat (1986) [107]	–	–	•	–	•	–	–	–	•	–	–	–	Voluntary motor control of the left quadriceps and spasm abolition occurred with eSCS	None reported
**2**	Dimitrijevic (1998) [30]	–	–	•	–	–	–	–	–	–	–	–	–	Rhythmic locomotor–like activity and stepping movement was recruited at frequencies of 25–100 Hz	None reported
**3**	Herman (2002) [32]	–	•	–	•	•	•	•	–	•	•	–	•	Immediate improvement in gait rhythm; speed, endurance, and metabolic responses gradually converged with/without eSCS at short distances, although performance with eSCS was superior at long distances	None reported
**4**	Cahart (2004) [31]	–	•	•	•	–	•	–	–	•	–	–	•	Reduction in sense of effort for OGW and doubling of walking speed initially. Walking speeds reached 0.35 m/s, with increased ambulation distance > 325m after several weeks of ground training.	High–frequency stimulation (100 Hz) produced discomfort.
**5**	Jilge (2004) [134]	–	–	•	–	–	–	–	–	–	–	–	–	eSCS at the lumbosacral region at 5–15 Hz initiates and sustains lower limb extension	None reported
**6**	Minassian (2004) [34]	–	–	•	–	–	–	–	–	–	–	–	–	Segmental selective recruitment of lower limb muscles, which is characteristic of posterior root stimulation. A 2.2 Hz stimulation recruited short–latency compound muscle action potentials, whilst 5–15 and 25–50 Hz stimulation elicited sustained tonic extension and rhythmic activity, respectively.	None reported
**7**	Ganley (2005) [135]	–	•	•	•	•	•	•	–	•	–	–	•	eSCS enabled patients to walk faster and further	None reported
**8**	Huang (2006) [33]	–	•	•	•	•	•	•	–	•	–	–	•	eSCS activates neural structures in the dorsal aspect of the spinal cord and facilitates gait–related muscle recruitment; eSCS improved walking speed, endurance, and reduced SoE	None recorded
**9**	Minassian (2007) [136]	–	–	•	–	–	–	–	–	–	–	–	–	5–15 Hz stimulation initiates lower limb extension; 25–50 Hz elicits alternating lower limb flexion/extension in supine individuals; and eSCS combined with assisted treadmill stepping increases excitability of lumbar cord networks and enhances stepping–like functional motor outputs	None reported
**10**	Harkema (2011) [22]	•	•	•	•	•	–	–	•	–	–	–	–	15 Hz stimulation was optimized for standing, and 30–40 Hz for stepping. Recovery of supraspinal control of some leg movements occurred after 7 months, but only during eSCS	None reported
**11**	Monshonkina (2012) [116]		•	•		•	•	–	–	–	–	–	–	Combination of eSCS with locomotor training led to stepping patterns characteristic of normal walking and tonic activity of muscles needed for body balance maintenance. With bipolar stimulation, thresholds of muscle responses were significantly lower than thresholds determined with monopolar stimulation.	None reported
**12**	Minassian (2013) [118]	•	•	•	–	–	–	–	–	–	–	–	–	eSCS produces rhythmic EMG activities without step–related sensory feedback. eSCS also immediately augmented EMG activities as generated by passive stepping alone, in addition to activating muscles that did not respond otherwise.	None reported
**13**	Angeli (2014) [35]	–	•	•	•	•	–	–	–	–	–	–	•	eSCS enables patients with complete paralysis process conceptual, auditory and visual input to regain relatively fine voluntary motor control of paralyzed muscles	None reported
**14**	Sayenko (2014) [62]	–	•	•	–	•	–	–	–	–	–	–	–	eSCS of rostral and caudal areas of the lumbar spinal cord led to selective topographical recruitment of proximal and distal leg muscles	None reported
**15**	Danner (2015) [137]	–	–	•	–	–	–	–	–	–	–	–	–	Rhythmic activity was generated in 7/10 subjects after stimulation; these rhythms demonstrated flexion and extension phases similar to those needed for locomotion	None reported
**16**	Hoefstoetter (2015) [138]	–	–	•	–	–	–	–	–	–	–	–	–	Repeated epidural stimulation of the lumbosacral spinal cord can generate rhythmic burst–like activity at 20–60 Hz	None reported
**17**	Rejc (2015) [36]	•	•	•	–	•	–	–	–	–	–	–	–	2 clinically sensory and motor complete participants could overground weight–bearing stand without external assistance; 2 clinically motor complete, sensory incomplete achieved hip extension with minimal assistance; caudal stimulation at higher frequencies (25–60 Hz) led to improve standing	Discomfort from abdominal contractions
**18**	Lu (2016) [117]	–	–	•	–	•	–	–	–	–	–	–	–	Cervical cord neuromodulation improves volitional hand motor function (grip and control) in individuals with chronic tetraplegia	None reported
**19**	Grahn (2017) [139]	•	•	•	–	•	–	–	–	–	–	–	–	eSCS with activity–specific training enabled (1) volitional control of task–specific muscle activity; (2) volitional control of rhythmic muscle activity to produce step–like movements while side–lying; (3) independent standing; and (4) voluntary control of step–like movements and rhythmic activity while in a vertical position with body weight partially supported	None noted
**20**	Rejc (2017) A [37]		•	•	–	•	–	–	–	–	–	•	–	eSCS with motor training led to ongoing neural adaptations that enabled a refined, task–specific activation pattern and movement duration in the absence of stimulation; re–emergence of muscle activation patterns sufficient for standing with independent knee and hip extension	None noted
**21**	Rejc (2017) B [38]		•	•	•	•	–	–	–	–	–	•	–	Standing improved in all participants after stand training, however, step training worsened standing ability in 3/4 participants	None noted
**22**	Angeli (2018) [39]	•	•	•	•	•	•	•	–	–	–	–	•	Intense locomotor treadmill training with body support and simultaneous eSCS led to independent walking and trunk stability in patients with complete spinal cord injury	Hip fracture during training (1); ankle oedema (1); drainage from surgery site (1)
**23**	Formento (2018) [40]	•	•	•	–	•	–	–	•	–	–	–	•	Proprioceptive information facilitates walking with eSCS. Thus, eSCS stimulation parameters that cancel proprioceptive information (continuous stimulation) prevent the modulation of reciprocal inhibitory networks involved in locomotion and reduces or abolishes the conscious perception of leg position	None reported
**24**	Gill (2018) [41]	•	•	•	•	•	–	•	•	–	•	–	•	Individuals with complete SCI have greater independence during body weight supported treadmill stepping if proprioceptive inputs are optimized through body weight support	None reported
**25**	Wagner (2018) [42]	–	•	•	•	•	•	•	•	–	–	•	•	eSCS re-established adaptive control of paralyzed muscles during overground walking within one week; spatiotemporal stimulation led to volitional control over walking and cycling.	None reported
**26**	Calvert (2019) [43]	–	–	•	–	•	–	–	–	–	–	–	–	eSCS–evoked motor responses guide intraoperative electrode placement to enable motor functions. Intentional control of step–like activity was achieved within first 5 days	None reported
**27**	Cheng (2019) [44]	–	–	•	–	–	–	–	–	–	–	–	–	During standing, eSCS activates an additional neural circuit, which is critical to, and improves, standing in SCI	None reported
**28**	Darrow (2019) [45]	–	–	•	–	•	–	–	–	–	–	–	–	Immediate restoration of volitional motor control with significant improvement in surface EMG during volitional control task with eSCS on	None noted
**29**	Gorgey (2020) [46]	•	–	•	–	•	•	•	–	–	–	–	–	After 24 sessions (12 weeks) of exoskeleton–assisted walking with eSCS, swing assistance decreased from 100% to 35%, accompanied by 573 unassisted steps (50% of total steps)	None noted
**30**	Penã Pino (2020) [47]	–	–	•	–	–	–	–	–	–	–	–	–	After eSCS, sustained volitional movement was achieved in 4/7 subjects even in the absence of stimulation; volitional power significantly increased with the ability to cycle without stimulation	None noted
**31**	Calvert (2021) [48]	–	–	–	–	•	–	–	–	–	–	–	–	eSCS combined with descending commands activate inhibitory inter–neuronal circuitry within spinal sensorimotor networks in SCI	None noted
**32**	Gill (2021) [141]	•	•	•	•	•	–	–	•	–	–	–	–	During eSCS–enabled BWST stepping, the knee extensors exhibited an increase in motor activation during trials in which stepping was passive compared to active or during trials in which 60% BWS was provided compared to 20% BWS	None noted
**33**	Ibánēz (2021) [49]	•	–	•	–	•	–	–	–	–	–	•	–	eSCS promotes both orderly (according to neuron size) and inverse trends of motor neuron recruitment, with the spinal networks involved in the generation of rhythmic activating favoring orderly recruitment trends	None noted
**34**	Linde (2021) [50]	–	–	–	•	•	–	–	–	–	–	–	•	eSCS (both on and off) combined with rehabilitation improved independence in stepping	None noted
**35**	Mesbah (2021) [51]	–	–	•	–	•	–	–	–	–	–	–	–	The region and position of lumbosacral enlargement covered by eSCS electrodes significantly correlates with the number of joints moved volitionally. All participants achieved lower extremity volitional motor control post eSCS and prior to any locomotor training	None noted
**36**	Gorgey (2022) [52]	•		•	–	–	–	–	–	–	–	–	–	Lumbosacral eSCS restored trunk control and maintained full standing in a person with complete paraplegia	Complete migration of left (outside the epidural space) and right ((from T11–12 to T12–L1) leads
**37**	Kandhari (2022) B [143]	•	•	–	–	•	–	–	–	–	–	–	•	AIS scores changed from A–C for 8 patients and A–D for 2 patients after 8 weeks, with 6 patients improving their functional level of injury by ≥1 segment. Significant improvement in lower extremity muscles were seen in all patients. Independence and comfort were seen during walking post–therapy	None reported
**38**	Rowald (2022) [53]	•	•	•	•	•	•	•	•	–	–	•	•	Activity–specific eSCS enabled standing, swimming, cycling, walking and control of trunk movements within 1 day; gait improvement and volitional motor control also occurred after 1 week post eSCS. Neurorehabilitation mediated the restoration of these locomotor activities in community settings	None reported
**39**	Smith (2022) [115]	•	–	•	–	•	–	–	–	–	–	•	–	Measures of spared spinal cord tissue significantly relate to standing outcomes with eSCS –– 7/11 subjects with spared spinal cord tissue achieved some knee independence	None reported
**40**	Boakye (2023) [108]	•	•	•	–	•	•	–	–	–	–	–	•	All participants achieved voluntary movement in the lower extremities after eSCS. There was a correlation between quality of life with training, functional improvement, and complications	Ileus (2); seroma (2); pain with stimulation (2); dehiscence (2); incision site erythema (1), drainage (1), and cellulitis (1); device infection (1); neurostimulator malposition requiring correction (1); and electrode malfunction (1)
**41**	Gorgey (2023) A [109]	•	–	•	•	•	•	•	–	–	–	•	–	eSCS enabled voluntary increased muscle activation and movement below the level of injury and promoted independence during exoskeleton–assisted walking. In one individual, eSCS enabled motor control (below the injury level), and independent standing and stepping	None reported
**42**	Gupta (2023) [111]	–	–	–	–	•	–	–	–	–	•	–	–	The patient could perform seated knee extension and hip flexion 2 days post–eSCS implantation. Leg spams and other unwanted movements were abolished following longer–term (3–4 times/week) stimulation	None reported
**43**	Hoover (2023) [112]	–	–	•	–	•	–	–	–	–	–	–	–	Following eSCS and conscious effort, all participants were able to pedal without motor assist; eSCS and effort were significantly correlated with maximum power production and distance pedaled. No association was found between volitional movement and patient factors (age, time since injury, and spinal cord atrophy)	None reported

A/I: assisted/independent standing/stepping; BWS: body weight support; EMG: electromyography; GA: gait analysis; GMA: general muscle activity; IWS: increased walking speed; OGW: overground walking; Prop: proprioception; SoE: sense of effort; Spas: spasticity; STS: sit–to–stand transition; TSW: treadmill step/walk.

**Table 4:** Successful locomotor function following eSCS.

# Author (Year)		Age	Years Since Injury	ASIA Score	Level	N	A/I	BWS	GMA	IWS	OGW	STS	TSW
1	Barolat (1986) [107]	22	0.75	C	C5	1	–	–	1/7	–	–	–	–
8	Huang (2006) [33]	43–48	3.5–8	C	C5–T8	2	–	0.6	2/2	2/2	2/2	–	•
11	Monshonkina (2012) [116]	22–58		2A, 1B, 1B/C	C5–L1	4	–	–	2/4	2/4	–	–	–
12	Minassian (2013) [118]	–		7A/B		7	0/2	0.6	–	–	–	–	–
18	Lu (2016) [117]	18–20	2–2.5	B	C5–C6	2	–	–	2/2	–	–	–	–
25	Wagner (2018) [42]	28–47	4–6	2C, 1D	C4–T8	3	–	0	3/3	3/3	2/3	•	•
26	Calvert (2019) [43]	26–37	3–6	A	T3, T6	2	–	–	2/2	–	–	–	–
28	Darrow (2019) [45]	48–52	5–10	A	T4, T8	2	–	–	2/2	–	–	–	–
29	Gorgey (2020) [46]	26	2	C	C5	1	0/1	–	1/1	1/1	•	–	–
31	Calvert (2021) [48]	26–36	3–6	A	T3, T6	2	–	–	2/2	–	–	–	–
34	Linde (2021) [50]	26–37	3–6	A	T3, T6	2	–	–	2/2	–	–	–	•
36	Gorgey (2022) [52]	25	3	A	T3	1	1/1		–	–	–	–	–
37	Kandhari (2022) [143]	21–51	0.3–2	A	T2–T12	10	10/10	–	10/10	–	–	–	•
38	Rowald (2022) [53]	29–41	1–9	2A, 1B	T3–T7	3	3/3	0	3/3	3/3	3/3	•	•
39	Smith (2022) [115]	21–45	2–9	6A, 5B	C2–T1	11	1/11	–	1/11	–	–	•	–
40	Boakye (2023) [108]	19–60	2–17	16A, 9B	C2–T5	25	8/25	•	25/25	2/8	–	–	•
41	Gorgey (2023) A [109]	–	6–9	1A, 1B	C8, T11	2	1/2	–	2/2	2/2	1/2	•	–
42	Gupta (2023) [111]	25	5		T6	1	–	–	1/1	–	–	–	–
43	Hoover (2023) [112]	26–58	3–17	6A, 1B	T4–T8	7	–	–	7/7	–	–	–	–

A/I: self-assisted/independent standing/stepping; BWS: body weight support; GMA: general muscle activity; IWS: increased walking speed; OGW: overground walking.BWS: defined success as reported % BWS needed with SCS for stepping; GMA: defined success based on subjective report in the article that this was achieved; A/I: defined success based on subjective report in the article that this was achieved.

**Table 5:** Assessment and outcomes of autonomic function following eSCS.

Author (Year)		Cardiovascular					Pulmonary			GI & Genitourinary				Key Findings	Complications
		BP	Orth	HR	CF	Pleth	PAW	PEFR	SPIR	Bowel	B/I	S&V	UD		
**1**	Katz (1991) [120]	–	–	–	–	–	–	–	–	–	–	–	•	Postoperative changes in the lower urinary tract function were noted in 6 patients. Urodynamic parameters did not change significantly following implantation in the remaining 17 patients	None reported
**2**	DiMarco (2006) [100]	–	–	–	–	–	•	•	–	–	–	–	–	Combined T9 + L1 stimulation led to airway pressure and expiratory flow rates observed with a normal subject: airway pressure increased to 90 cm H_2_O (T9), 82 cm H_2_O (L1) and 132 cm H_2_O (T9 + L1); peak expiratory flow rate also increased to 6.4 L/s (T9), 5.0 L/s (L1), and 7.4 L/s (T9 + L1).	None reported
**3**	DiMarco (2009) [101]	–	–	–	–	–	•	•	–	–	–	–	–	Supramaximal SCS led to increases in both mean maximum peak airflow rates, from mean 1.86 ± 0.17 L/s at baseline to 8.6 ± 1.8 L/s (mean ± SE), and airway pressure from 22.4 ± 1.18 cm H_2_O at baseline to 137 ± 30 cm H_2_O (mean ± SE).	One non–functional lead (9); breakdown and infection (1); temporary asymptomatic AD (3)
**4**	Harkema (2011) [22]	–	–	–	–	–	–	–	–	–	–	•	–	eSCS with training led to functional gains in bladder and sexual function–the patient was able to voluntarily void with minimal residual volume of urine after previously having no voluntary bladder contraction.	None reported
**5**	Aslan (2018) [141]	•	•	•	–	•	–	–	–	–	–	–	–	eSCS applied while supine and standing resulted in increased arterial BP in individuals with SCI–induced cardiovascular deficits	None reported
**6**	DiMarco (2018) [102]	–	–	–	–	–	•	•	–	–	–	–	–	Spontaneous maximum airway pressure increased from 20 cm H_2_O to 61 and 86 cmH_2_O for FRC and TLC, respectively (monopolar stimulation, T9) and to 84 and 103 cmH_2_O for FRC and TLC, respectively (bipolar stimulation, T9 + T11); the subject also experienced greater sense in raising secretions with eSCS and no longer required other methods of secretion management	Temporary asymptomatic AD that resolved after 5–6 weeks
**7**	Harkema (2018) A [78]	•	•	•	–	•	–	–	–	–	–	–	–	Dorsal lumbosacral eSCS can effectively and safely activate mechanisms to elevate BP to normal ranges from a chronic hypotensive state in humans with severe SCI with individual–specific cardiovascular eSCS	None reported
**8**	Harkema (2018) B [132]	•	•	•	–	•	–	–	–	–	–	–	–	Orthostatic hypotension resolved with cardiovascular eSCS and after daily eSCS training without stimulation	None reported
**9**	Herrity (2018) [130]	–	–	–	–	–	–	–	–	–	–	•	•	Optimized parameters yielded lowest post–void residual volume and also improved reflexive voiding efficiency	None reported
**10**	Walter (2018) [126]	–	–	–	–	–	–	–	–	–	–	–	•	eSCS significantly reduced the time needed for bowel management and modulated detrusor pressure and external sphincter/pelvic floor muscle tone.	None reported
**11**	West (2018) [79]	•	•		•	•	–	–	–	–	–	–	–	The stimulation resolved orthostatic hypotension and related symptoms through action on preventing reduced MCA blood flow; eSCS also improved cardiac function	None reported
**12**	Darrow (2019) [45]	•	•	•	•	–	–	–	–	•	•	•	–	eSCS led to restoration of cardiovascular function, orgasm, volitional urination, and improved surface EMG power during a volitional control task with eSCS on	None reported
**13**	Nightingale (2019) [130]	•	•	–	•	–	–	–	–	–	–	–	–	eSCS parameters optimized to facilitate motor function can also modulate cardiovascular function (increasing or maintaining arterial BP at rest or in response to an orthostatic challenge, respectively)	None reported
**14**	Terson de Paleville (2019) [122]	•	–	•	–	–	–	–	–	–	–	–	–	Combined eSCS and task–specific training improves cardiovascular fitness and body composition (reduces percentage fat, particularly android fat and android/gynoid ratio)	None reported
**15**	DiMarco (2020) [102]	–	–	–	–	–	•	•	–	–	–	–	–	SCS improved both expiratory muscle function (maximum expiratory pressure and cough restoration) and inspiratory muscle function (inspiratory capacity and maximum inspiratory pressure) after 20 weeks following daily stimulation	None reported
**16**	Beck (2021) [120]	–	–	–	–	–	–	–	–	–	•	•	•	eSCS optimized for locomotion negatively impacted neurogenic bladder functionality, leading to increase in episodes of urinary incontinence with worsening bladder compliance and pressures. One participant showed increase in lean body mass	None reported
**17**	DiMarco (2021) [123]	–	–	–	–	–	•	•	•	•	–	–	–	Optimized eSCS parameters for maximum airway pressure generation improves bowel movements and quality of life	None reported
**18**	Herrity (2021) [121]	•	–	•	–	•		–	–	–	–	•	•	Bladder storage parameters were significantly improved at post–training and at follow–up. Elevated BP during bladder extension, which is characteristic of AD, was however not attenuated	None reported
**19**	Kandhari (2022) A [114]	–	–	–	–	–	•	–	•	–	–	–	–	eSCS at T2–T5 improved pulmonary function by increasing inspired volume, and promoting pulmonary dependence from mechanical ventilation to pressure support	None reported
**20**	Squair (2021) [98]	•	•	•	–	•	–	–	–	–	–	–	–	eSCS activates the sympathetic circuitry by increasing sympathetic nerve activity and normalizing circulating NE levels. Real–time hemodynamic stabilization during orthostatic challenges was seen with eSCS, which reduces the burden of orthostatic hypotension with long–term use	None reported
**21**	Herrity (2022) [142]	•	–	•	–	–	–	–	–	–	•	•	•	eSCS reduces incidences of urinary incontinence and provides a means for mitigating AD associated with bladder distension	None reported
**22**	Boakye (2023) [108]	•	–	–	–	–	–	–	–	–	–	–	–	All participants achieved SBP regulation within 110–120 mmHg and were able to integrate the eSCS into their daily lives; no worsening of bladder function was seen	Same as reported for motor outcomes
**23**	Gorgey (2023) B [110]	•	•	•	–	–	–	–	–	–	–	–	–	Post eSCS implantation cardiovascular autonomic control was enhanced during transitions from a supine position to a 45–degree head–up–tilt (orthostatic challenge)	None reported
**24**	Samijema (2023) [113]	•	–	•	–	–	–	–	–	–	–	–	–	Lumbosacral eSCS reduces elevation in BP during bowel procedures, preventing AD	None reported

AD: autonomic dysreflexia; B/I: bladder incontinence; BP: blood pressure; CF: cardiac function; HR: heart rate; Orth: BP regulation during orthostasis; NE: norepinephrine; PAW; airway pressure; Pleth: plethysmography; PEFR: peak expiratory flow rate; S&V: bladder storage and voiding; SBP: systolic blood pressure; SPIR: spirometry; UD: urodynamics.

### 5.5. Limitations and Future Perspectives

Systematic reviews contain two sources of limitations: those inherent to the review itself (the method) and those inherent to the included articles (the data). Our methodology aimed to provide a comprehensive review of all articles published on the topic of eSCS in SCI. One limitation of this approach is the multiple published articles from the same study groups, which may have led to overestimating the total number of patients who have undergone eSCS as well as its apparent effect size. To minimize overestimating the reported impact of eSCS, we excluded articles with overlapping cohorts reporting locomotor outcomes. This, however, was limited by individual articles identifying overlapping patients, and may have led to a sampling bias. Further, we manually extracted qualitative assessments from included articles. This may lead to human error, which we aimed to minimize by adjudication of included articles by our senior authors.

Our results and conclusions should be understood within the context of this emerging field and are limited by the small number of research participants and research groups currently investigating eSCS in patients with SCI. A further limitation is that most articles were case reports or case series, including a few prospective case series, with no case–control, cohort, or randomized control trials. Therefore, the results of our review are limited to only level 4 evidence. However, the majority of published research reported analyses from currently ongoing well-designed studies, which will provide higher quality evidence in the future. Another limitation of this review is the lack of reporting of motor function with the well-validated ASIA scale. Although ASIA grade was typically reported before eSCS, a detailed assessment of motor strength of individual muscle groups was lacking in the majority of published articles. The 6-point assessment of motor function is a common tool that physicians and health professionals across various disciplines are well familiar with. It is an important outcome of eSCS effect on motor function crucial for future research.

Several gaps in the literature present opportunities for highly impactful future research. One critical challenge lies in characterizing the specific patient profiles that would derive the most benefit from eSCS. An essential step forward involves discerning whether eSCS universally benefits all chronic SCI patients or primarily those with anatomically incomplete SCI. Presently, clinical practice does not mandate neuroimaging assessments for chronic SCI patients. We think that it will become imperative to characterize the remaining corticospinal and other descending and ascending tracts at the injury site in potential eSCS candidates. Customizing eSCS interventions based on the ASIA classification, particularly for ASIA B patients who may exhibit improved outcomes due to preserved sensory cues, is of paramount importance.

The role of physical therapy and neurorehabilitation within the context of eSCS cannot be overstated. Determining the type, duration, and necessity of physical therapy for eSCS candidates is pivotal for optimizing outcomes. Access to specialized neurorehabilitation both before and after eSCS implantation significantly influences the success of this treatment, particularly concerning voluntary motor control and independent locomotion.

Optimizing stimulation parameters is crucial for achieving the best possible outcomes with eSCS. This entails exploring a wide range of frequencies (ranging from 0.2 to 400 Hz), pulse widths (ranging from 150 to 100 μsec), and amplitudes (ranging from 0.1 to 40 V/0.1 to 15 mA). A comprehensive understanding of the mechanisms underlying eSCS and its effects on both motor and autonomic function will be invaluable. Furthermore, the precise placement of electrodes plays a pivotal role in determining the success of the intervention. Tailoring electrode placement for specific outcomes, such as T9–T11 for pulmonary functions, T11–L1 for volitional motor control, and L1–S1 for genitourinary functions, is essential.

Our analysis underscores the predominance of case reports and small case series in the current literature, signaling a gap in published well-designed prospective studies. Addressing this limitation necessitates interdisciplinary multi-center research initiatives that could significantly enhance the evidence base for eSCS as a treatment for SCI.

### 5.6. Recommendations

To maximize the effectiveness of epidural spinal cord stimulation as a treatment for spinal cord injury, we suggest several key recommendations. Firstly, a patient-centric approach is paramount, tailoring treatment plans and recovery expectations to individual patients based on their specific SCI characteristics, including ASA grade and injury level. Prioritizing task-specific training is critical to optimizing outcomes, as is ensuring precise electrode placement, guided by an understanding of the underlying mechanisms. Different spinal level placements can yield varied outcomes for treating symptoms and enhancing overall function. There needs to be a method for parameter optimization given the wide variation in stimulation parameters. Physical therapy (PT) and intensive neurorehabilitation play pivotal roles, and despite their time-intensive nature, they should not be short-changed. Furthermore, there are considerable challenges associated with regaining advanced movements like walking, so this research should be conducted in major academic centers. Vigilant monitoring for adverse effects by physicians is essential. 

## 6. Conclusions

Epidural SCS has emerged as a novel method of improving motor, autonomic, and genitourinary function in patients with paralysis secondary to SCI. Multiple articles report recovery of function in patients with chronic SCI when appropriate stimulation settings are established. The included research demonstrates that the field of restoring motor, autonomic, and genitourinary function with eSCS is still in its infancy and carries a tremendous potential to impact quality of life of patients living with SCI and their caregivers. This creates an exciting opportunity for future research to investigate the efficacy, mechanisms, and potential challenges of this intervention. By refining patient selection, tailoring interventions, optimizing stimulation parameters, advancing translational research, and fostering collaborative, high-quality studies, we can pave the way for a brighter future for individuals with SCI.

## Data Availability

Not applicable.

## Supplementary Materials

The following supporting information can be downloaded at: https://www.mdpi.com/article/10.3390/jcm13041090/s1, Supplementary Table S1: ROBINS-I Risk of Bias Analysis [144].

## References

[1] National Spinal Cord Injury Statistical Center (2020). Facts and Figures at a Glance.

[2] Lo J., Chan L., Flynn S. (2021). A Systematic Review of the Incidence, Prevalence, Costs, and Activity and Work Limitations of Amputation, Osteoarthritis, Rheumatoid Arthritis, Back Pain, Multiple Sclerosis, Spinal Cord Injury, Stroke, and Traumatic Brain Injury in the United States: A 2019 Update. Arch. Phys. Med. Rehabil..

[3] Roberts T.T., Leonard G.R., Cepela D.J. (2017). Classifications in Brief: American Spinal Injury Association (ASIA) Impairment Scale. Clin. Orthop. Relat. Res..

[4] Sezer N., Akkuş S., Uğurlu F.G. (2015). Chronic complications of spinal cord injury. World J. Orthop..

[5] Hachem L.D., Ahuja C.S., Fehlings M.G. (2017). Assessment and management of acute spinal cord injury: From point of injury to rehabilitation. J. Spinal Cord Med..

[6] Russo G.S., Mangan J.J., Galetta M.S., Boody B., Bronson W., Segar A., Kepler C.K., Kurd M.F., Hilibrand A.S., Vaccaro A.R. (2020). Update on Spinal Cord Injury Management. Clin. Spine Surg..

[7] Truchon C., Fallah N., Santos A., Vachon J., Noonan V.K., Cheng C.L. (2017). Impact of Therapy on Recovery during Rehabilitation in Patients with Traumatic Spinal Cord Injury. J. Neurotrauma.

[8] Arazpour M., Bani M.A., Hutchins S.W., Jones R.K. (2013). The physiological cost index of walking with mechanical and powered gait orthosis in patients with spinal cord injury. Spinal Cord.

[9] Mekki M., Delgado A.D., Fry A., Putrino D., Huang V. (2018). Robotic Rehabilitation and Spinal Cord Injury: A Narrative Review. Neurotherapeutics.

[10] Shinozaki M., Nagoshi N., Nakamura M., Okano H. (2021). Mechanisms of Stem Cell Therapy in Spinal Cord Injuries. Cells.

[11] Guo J.S., Qian C.H., Ling E.A., Zeng Y.S. (2014). Nanofiber scaffolds for treatment of spinal cord injury. Curr. Med. Chem..

[12] Li X., Liu D., Xiao Z., Zhao Y., Han S., Chen B., Dai J. (2019). Scaffold-facilitated locomotor improvement post complete spinal cord injury: Motor axon regeneration versus endogenous neuronal relay formation. Biomaterials.

[13] Zou Y. (2021). Targeting axon guidance cues for neural circuit repair after spinal cord injury. J. Cereb. Blood Flow Metab..

[14] Ladenbauer J., Minassian K., Hofstoetter U.S., Dimitrijevic M.R., Rattay F. (2010). Stimulation of the human lumbar spinal cord with implanted and surface electrodes: A computer simulation study. IEEE Trans. Neural Syst. Rehabil. Eng..

[15] Capogrosso M., Wenger N., Raspopovic S., Musienko P., Beauparlant J., Bassi Luciani L., Courtine G., Micera S. (2013). A computational model for epidural electrical stimulation of spinal sensorimotor circuits. J. Neurosci..

[16] Van den Brand R., Heutschi J., Barraud Q., DiGiovanna J., Bartholdi K., Huerlimann M., Friedli L., Vollenweider I., Moraud E.M., Duis S. (2012). Restoring voluntary control of locomotion after paralyzing spinal cord injury. Science.

[17] Shah P.K., Sureddi S., Alam M., Zhong H., Roy R.R., Edgerton V.R., Gerasimenko Y. (2016). Unique Spatiotemporal Neuromodulation of the Lumbosacral Circuitry Shapes Locomotor Success after Spinal Cord Injury. J. Neurotrauma.

[18] Melzack R., Wall P. (1965). Pain mechanisms: A new theory. Science.

[19] Wall P.D., Sweet W.H. (1967). Temporary abolition of pain in man. Science.

[20] Shealy C.N., Taslitz N., Mortimer J.T., Becker D.P. (1967). Electrical inhibition of pain: Experimental evaluation. Anesth. Analg..

[21] Cook A.W., Weinstein S.P. (1973). Chronic dorsal column stimulation in multiple sclerosis. Prelim. Rep. N. Y. State J. Med..

[22] Harkema S., Gerasimenko Y., Hodes J., Burdick J., Angeli C., Chen Y., Ferreira C., Willhite A., Rejc E., Grossman R.G. (2011). Effect of epidural stimulation of the lumbosacral spinal cord on voluntary movement, standing, and assisted stepping after motor complete paraplegia: A case study. Lancet.

[23] Siegfried J., Lazorthes Y., Broggi G. (1981). Electrical spinal cord stimulation for spastic movement disorders. Appl. Neurophysiol..

[24] Richardson R.R., McLone D.G. (1978). Percutaneous epidural neurostimulation for paraplegic spasticity. Surg. Neurol..

[25] Dimitrijevic M.M., Dimitrijevic M.R., Illis L.S., Nakajima K., Sharkey P.C., Sherwood A.M. (1986). Spinal cord stimulation for the control of spasticity in patients with chronic spinal cord injury: I. Clinical observations. Cent. Nerv. Syst. Trauma.

[26] Pinter M.M., Gerstenbrand F., Dimitrijevic M.R. (2000). Epidural electrical stimulation of posterior structures of the human lumbosacral cord: 3. Control of spasticity. Spinal Cord.

[27] Sherrington C.S. (1906). Observations on the scratch-reflex in the spinal dog. J. Physiol..

[28] Grillner S., Zangger P. (1975). How detailed is the central pattern generation for locomotion?. Brain Res..

[29] Bussel B., Roby-Brami A., Néris O.R., Yakovleff A. (1996). Evidence for a spinal stepping generator in man. Paraplegia.

[30] Dimitrijevic M.R., Gerasimenko Y., Pinter M.M. (1998). Evidence for a spinal central pattern generator in humans. Ann. N. Y. Acad. Sci..

[31] Carhart M.R., He J., Herman R., D’Luzansky S., Willis W.T. (2004). Epidural spinal-cord stimulation facilitates recovery of functional walking following incomplete spinal-cord injury. IEEE Trans. Neural Syst. Rehabil. Eng..

[32] Herman R., He J., D’Luzansky S., Willis W., Dilli S. (2002). Spinal cord stimulation facilitates functional walking in a chronic, incomplete spinal cord injured. Spinal Cord.

[33] Huang H., He J., Herman R., Carhart M.R. (2006). Modulation effects of epidural spinal cord stimulation on muscle activities during walking. IEEE Trans. Neural Syst. Rehabil. Eng..

[34] Minassian K., Jilge B., Rattay F., Pinter M.M., Binder H., Gerstenbrand F., Dimitrijevic M.R. (2004). Stepping-like movements in humans with complete spinal cord injury induced by epidural stimulation of the lumbar cord: Electromyographic study of compound muscle action potentials. Spinal Cord.

[35] Angeli C.A., Edgerton V.R., Gerasimenko Y.P., Harkema S.J. (2014). Altering spinal cord excitability enables voluntary movements after chronic complete paralysis in humans. Brain.

[36] Rejc E., Angeli C., Harkema S. (2015). Effects of Lumbosacral Spinal Cord Epidural Stimulation for Standing after Chronic Complete Paralysis in Humans. PLoS ONE.

[37] Rejc E., Angeli C.A., Atkinson D., Harkema S.J. (2017). Motor recovery after activity-based training with spinal cord epidural stimulation in a chronic motor complete paraplegic. Sci. Rep..

[38] Rejc E., Angeli C.A., Bryant N., Harkema S.J. (2017). Effects of Stand and Step Training with Epidural Stimulation on Motor Function for Standing in Chronic Complete Paraplegics. J. Neurotrauma.

[39] Angeli C.A., Boakye M., Morton R.A., Vogt J., Benton K., Chen Y., Ferreira C.K., Harkema S.J. (2018). Recovery of over-Ground Walking after Chronic Motor Complete Spinal Cord Injury. N. Engl. J. Med..

[40] Formento E., Minassian K., Wagner F., Mignardot J.B., Le Goff-Mignardot C.G., Rowald A., Bloch J., Micera S., Capogrosso M., Courtine G. (2018). Electrical spinal cord stimulation must preserve proprioception to enable locomotion in humans with spinal cord injury. Nat. Neurosci..

[41] Gill M.L., Grahn P.J., Calvert J.S., Linde M.B., Lavrov I.A., Strommen J.A., Beck L.A., Sayenko D.G., Van Straaten M.G., Drubach D.I. (2018). Neuromodulation of lumbosacral spinal networks enables independent stepping after complete paraplegia. Nat. Med..

[42] Wagner F.B., Mignardot J.B., Le Goff-Mignardot C.G., Demesmaeker R., Komi S., Capogrosso M., Rowald A., Seáñez I., Caban M., Pirondini E. (2018). Targeted neurotechnology restores walking in humans with spinal cord injury. Nature.

[43] Calvert J.S., Grahn P.J., Strommen J.A., Lavrov I.A., Beck L.A., Gill M.L., Linde M.B., Brown D.A., Van Straaten M.G., Veith D.D. (2019). Electrophysiological Guidance of Epidural Electrode Array Implantation over the Human Lumbosacral Spinal Cord to Enable Motor Function after Chronic Paralysis. J. Neurotrauma.

[44] Cheng R., Sui Y., Sayenko D., Burdick J.W. (2019). Motor Control after Human SCI through Activation of Muscle Synergies under Spinal Cord Stimulation. IEEE Trans. Neural Syst. Rehabil. Eng..

[45] Darrow D., Balser D., Netoff T.I., Krassioukov A., Phillips A., Parr A., Samadani U. (2019). Epidural Spinal Cord Stimulation Facilitates Immediate Restoration of Dormant Motor and Autonomic Supraspinal Pathways after Chronic Neurologically Complete Spinal Cord Injury. J. Neurotrauma.

[46] Gorgey A.S., Gill S., Holman M.E., Davis J.C., Atri R., Bai O., Goetz L., Lester D.L., Trainer R., Lavis T.D. (2020). The feasibility of using exoskeletal-assisted walking with epidural stimulation: A case report study. Ann. Clin. Transl. Neurol..

[47] Peña Pino I., Hoover C., Venkatesh S., Ahmadi A., Sturtevant D., Patrick N., Freeman D., Parr A., Samadani U., Balser D. (2020). Long-Term Spinal Cord Stimulation after Chronic Complete Spinal Cord Injury Enables Volitional Movement in the Absence of Stimulation. Front. Syst. Neurosci..

[48] Calvert J.S., Gill M.L., Linde M.B., Veith D.D., Thoreson A.R., Lopez C., Lee K.H., Gerasimenko Y.P., Edgerton V.R., Lavrov I.A. (2021). Voluntary Modulation of Evoked Responses Generated by Epidural and Transcutaneous Spinal Stimulation in Humans with Spinal Cord Injury. J. Clin. Med..

[49] Ibáñez J., Angeli C.A., Harkema S.J., Farina D., Rejc E. (2021). Recruitment order of motor neurons promoted by epidural stimulation in individuals with spinal cord injury. J. Appl. Physiol..

[50] Linde M.B., Thoreson A.R., Lopez C., Gill M.L., Veith D.D., Hale R.F., Calvert J.S., Grahn P.J., Fautsch K.J., Sayenko D.G. (2021). Quantitative Assessment of Clinician Assistance during Dynamic Rehabilitation Using Force Sensitive Resistors. Front. Rehabil. Sci..

[51] Mesbah S., Ball T., Angeli C., Rejc E., Dietz N., Ugiliweneza B., Harkema S., Boakye M. (2021). Predictors of volitional motor recovery with epidural stimulation in individuals with chronic spinal cord injury. Brain.

[52] Gorgey A.S., Gouda J.J. (2022). Single Lead Epidural Spinal Cord Stimulation Targeted Trunk Control and Standing in Complete Paraplegia. J. Clin. Med..

[53] Rowald A., Komi S., Demesmaeker R., Baaklini E., Hernandez-Charpak S.D., Paoles E., Montanaro H., Cassara A., Becce F., Lloyd B. (2022). Activity-dependent spinal cord neuromodulation rapidly restores trunk and leg motor functions after complete paralysis. Nat. Med..

[54] Logé D., De Coster O., Washburn S. (2012). Technological innovation in spinal cord stimulation: Use of a newly developed delivery device for introduction of spinal cord stimulation leads. Neuromodulation.

[55] Pahapill P.A. (2014). Surgical paddle-lead placement for screening trials of spinal cord stimulation. Neuromodulation.

[56] Lacroix-Ouellette P., Dubuc R. (2023). Brainstem neural mechanisms controlling locomotion with special reference to basal vertebrates. Front. Neural Circuits.

[57] Kiehn O. (2006). Locomotor circuits in the mammalian spinal cord. Annu. Rev. Neurosci..

[58] Chalif J.I., Martínez-Silva M.L., Pagiazitis J.G., Murray A.J., Mentis G.Z. (2022). Control of mammalian locomotion by ventral spinocerebellar tract neurons. Cell.

[59] Whelan P., Bonnot A., O’Donovan M.J. (2000). Properties of rhythmic activity generated by the isolated spinal cord of the neonatal mouse. J. Neurophysiol..

[60] Laliberte A.M., Goltash S., Lalonde N.R., Bui T.V. (2019). Propriospinal Neurons: Essential Elements of Locomotor Control in the Intact and Possibly the Injured Spinal Cord. Front. Cell. Neurosci..

[61] Courtine G., Gerasimenko Y., van den Brand R., Yew A., Musienko P., Zhong H., Song B., Ao Y., Ichiyama R.M., Lavrov I. (2009). Transformation of nonfunctional spinal circuits into functional states after the loss of brain input. Nat. Neurosci..

[62] Sayenko D.G., Angeli C., Harkema S.J., Edgerton V.R., Gerasimenko Y.P. (2014). Neuromodulation of evoked muscle potentials induced by epidural spinal-cord stimulation in paralyzed individuals. J. Neurophysiol..

[63] Guertin P., Angel M.J., Perreault M.C., McCrea D.A. (1995). Ankle extensor group I afferents excite extensors throughout the hindlimb during fictive locomotion in the cat. J. Physiol..

[64] Stein R.B., Capaday C. (1988). The modulation of human reflexes during functional motor tasks. Trends Neurosci..

[65] Harkema S.J., Hurley S.L., Patel U.K., Requejo P.S., Dobkin B.H., Edgerton V.R. (1997). Human lumbosacral spinal cord interprets loading during stepping. J. Neurophysiol..

[66] Cappellini G., Ivanenko Y.P., Dominici N., Poppele R.E., Lacquaniti F. (2010). Migration of motor pool activity in the spinal cord reflects body mechanics in human locomotion. J. Neurophysiol..

[67] Courtine G., Song B., Roy R.R., Zhong H., Herrmann J.E., Ao Y., Qi J., Edgerton V.R., Sofroniew M.V. (2008). Recovery of supraspinal control of stepping via indirect propriospinal relay connections after spinal cord injury. Nat. Med..

[68] Martinez M., Delivet-Mongrain H., Leblond H., Rossignol S. (2012). Effect of locomotor training in completely spinalized cats previously submitted to a spinal hemisection. J. Neurosci..

[69] Lavrov I., Courtine G., Dy C.J., van den Brand R., Fong A.J., Gerasimenko Y., Zhong H., Roy R.R., Edgerton V.R. (2008). Facilitation of stepping with epidural stimulation in spinal rats: Role of sensory input. J. Neurosci..

[70] Lavrov I., Gerasimenko Y., Burdick J., Zhong H., Roy R.R., Edgerton V.R. (2015). Integrating multiple sensory systems to modulate neural networks controlling posture. J. Neurophysiol..

[71] Takeoka A., Vollenweider I., Courtine G., Arber S. (2014). Muscle spindle feedback directs locomotor recovery and circuit reorganization after spinal cord injury. Cell.

[72] Takeoka A., Arber S. (2019). Functional Local Proprioceptive Feedback Circuits Initiate and Maintain Locomotor Recovery after Spinal Cord Injury. Cell Rep..

[73] Forssberg H., Grillner S., Halbertsma J. (1980). The locomotion of the low spinal cat. I. Coordination within a hindlimb. Acta Physiol. Scand..

[74] Baumbauer K.M., Hoy K.C., Huie J.R., Hughes A.J., Woller S.A., Puga D.A., Setlow B., Grau J.W. (2008). Timing in the absence of supraspinal input I: Variable, but not fixed, spaced stimulation of the sciatic nerve undermines spinally-mediated instrumental learning. Neuroscience.

[75] Grillner S., El Manira A. (2020). Current Principles of Motor Control, with Special Reference to Vertebrate Locomotion. Physiol. Rev..

[76] Tesio L., Scarano S. (2021). Ground Walking in Chronic Complete Spinal Cord Injury: Does Epidural Stimulation Allow “Awakening” of Corticospinal Circuits? A Wide-Ranging Epistemic Criticism. Am. J. Phys. Med. Rehabil..

[77] Harkema S.J., Wang S., Angeli C.A., Chen Y., Boakye M., Ugiliweneza B., Hirsch G.A. (2018). Normalization of Blood Pressure with Spinal Cord Epidural Stimulation after Severe Spinal Cord Injury. Front. Hum. Neurosci..

[78] West C.R., Phillips A.A., Squair J.W., Williams A.M., Walter M., Lam T., Krassioukov A.V. (2018). Association of Epidural Stimulation with Cardiovascular Function in an Individual with Spinal Cord Injury. JAMA Neurol..

[79] Collins H.L., DiCarlo S.E. (2002). TENS attenuates response to colon distension in paraplegic and quadriplegic rats. Am. J. Physiol. Heart Circ. Physiol..

[80] Sachdeva R., Nightingale T.E., Pawar K., Kalimullina T., Mesa A., Marwaha A., Williams A.M.M., Lam T., Krassioukov A.V. (2021). Noninvasive Neuroprosthesis Promotes Cardiovascular Recovery after Spinal Cord Injury. Neurotherapeutics.

[81] Legg Ditterline B.E., Aslan S.C., Wang S., Ugiliweneza B., Hirsch G.A., Wecht J.M., Harkema S. (2021). Restoration of autonomic cardiovascular regulation in spinal cord injury with epidural stimulation: A case series. Clin. Auton. Res..

[82] DiMarco A.F., Romaniuk J.R., Kowalski K.E., Supinski G. (1999). Pattern of expiratory muscle activation during lower thoracic spinal cord stimulation. J. Appl. Physiol..

[83] DiMarco A.F., Kowalski K.E. (2013). Activation of inspiratory muscles via spinal cord stimulation. Respir. Physiol. Neurobiol..

[84] Gonzalez-Rothi E.J., Streeter K.A., Hanna M.H., Stamas A.C., Reier P.J., Baekey D.M., Fuller D.D. (2017). High-frequency epidural stimulation across the respiratory cycle evokes phrenic short-term potentiation after incomplete cervical spinal cord injury. J. Neurophysiol..

[85] Jensen V.N., Alilain W.J., Crone S.A. (2020). Role of Propriospinal Neurons in Control of Respiratory Muscles and Recovery of Breathing Following Injury. Front. Syst. Neurosci..

[86] Nair J., Bezdudnaya T., Zholudeva L.V., Detloff M.R., Reier P.J., Lane M.A., Fuller D.D. (2017). Histological identification of phrenic afferent projections to the spinal cord. Respir. Physiol. Neurobiol..

[87] Nair J., Streeter K.A., Turner S.M.F., Sunshine M.D., Bolser D.C., Fox E.J., Davenport P.W., Fuller D.D. (2017). Anatomy and physiology of phrenic afferent neurons. J. Neurophysiol..

[88] Baumann S.B., Wozny D.R., Kelly S.K., Meno F.M. (1997). The electrical conductivity of human cerebrospinal fluid at body temperature. IEEE Trans. Biomed. Eng..

[89] Tawfik V.L., Chang S.Y., Hitti F.L., Roberts D.W., Leiter J.C., Jovanovic S., Lee K.H. (2010). Deep brain stimulation results in local glutamate and adenosine release: Investigation into the role of astrocytes. Neurosurgery.

[90] Karnup S.V., de Groat W.C. (2020). Propriospinal Neurons of L3–L4 Segments Involved in Control of the Rat External Urethral Sphincter. Neuroscience.

[91] de Groat W.C., Yoshimura N. (2009). Afferent nerve regulation of bladder function in health and disease. Handb. Exp. Pharmacol..

[92] Chéhensse C., Facchinetti P., Bahrami S., Andrey P., Soler J.M., Chrétien F., Bernabe J., Clement P., Denys P., Giuliano F. (2017). Human spinal ejaculation generator. Ann. Neurol..

[93] Boggs J.W., Wenzel B.J., Gustafson K.J., Grill W.M. (2006). Frequency-dependent selection of reflexes by pudendal afferents in the cat. J. Physiol..

[94] de Groat W.C., Ryall R.W. (1969). Reflexes to sacral parasympathetic neurones concerned with micturition in the cat. J. Physiol..

[95] Tai C., Wang J., Wang X., de Groat W.C., Roppolo J.R. (2007). Bladder inhibition or voiding induced by pudendal nerve stimulation in chronic spinal cord injured cats. Neurourol. Urodyn..

[96] Schurch B., Reilly I., Reitz A., Curt A. (2003). Electrophysiological recordings during the peripheral nerve evaluation (PNE) test in complete spinal cord injury patients. World J. Urol..

[97] Burns M., Solinsky R. (2022). Toward rebalancing blood pressure instability after spinal cord injury with spinal cord electrical stimulation: A mini review and critique of the evolving literature. Auton. Neurosci..

[98] Squair J.W., Gautier M., Mahe L., Soriano J.E., Rowald A., Bichat A., Cho N., Anderson M.A., James N.D., Gandar J. (2021). Neuroprosthetic baroreflex controls haemodynamics after spinal cord injury. Nature.

[99] DiMarco A.F., Kowalski K.E., Geertman R.T., Hromyak D.R. (2006). Spinal cord stimulation: A new method to produce an effective cough in patients with spinal cord injury. Am. J. Respir. Crit. Care Med..

[100] DiMarco A.F., Kowalski K.E., Geertman R.T., Hromyak D.R., Frost F.S., Creasey G.H., Nemunaitis G.A. (2009). Lower thoracic spinal cord stimulation to restore cough in patients with spinal cord injury: Results of a National Institutes of Health-Sponsored clinical trial. Part II: Clinical outcomes. Arch. Phys. Med. Rehabil..

[101] DiMarco A.F., Geertman R.T., Tabbaa K., Polito R.R., Kowalski K.E. (2018). Case report: Minimally invasive method to activate the expiratory muscles to restore cough. J. Spinal Cord Med..

[102] DiMarco A.F., Geertman R.T., Tabbaa K., Nemunaitis G.A., Kowalski K.E. (2020). Restoration of cough via spinal cord stimulation improves pulmonary function in tetraplegics. J. Spinal Cord Med..

[103] Donnelly D.F., Cohen M.I., Sica A.L., Zhang H. (1985). Responses of early and late onset phrenic motoneurons to lung inflation. Respir. Physiol..

[104] Sieck G.C. (1994). Physiological effects of diaphragm muscle denervation and disuse. Clin. Chest Med..

[105] DiMarco A.F., Kowalski K.E. (2009). High-frequency spinal cord stimulation of inspiratory muscles in dogs: A new method of inspiratory muscle pacing. J. Appl. Physiol..

[106] Page M.J., McKenzie J.E., Bossuyt P.M., Boutron I., Hoffmann T.C., Mulrow C.D., Shamseer L., Tetzlaff J.M., Akl E.A., Brennan S.E. (2021). The PRISMA 2020 statement: An updated guideline for reporting systematic reviews. BMJ.

[107] Barolat G., Myklebust J.B., Wenninger W. (1986). Enhancement of voluntary motor function following spinal cord stimulation—Case study. Appl. Neurophysiol..

[108] Boakye M., Ball T., Dietz N., Sharma M., Angeli C., Rejc E., Kirshblum S., Forrest G., Arnold F.W., Harkema S. (2023). Spinal cord epidural stimulation for motor and autonomic function recovery after chronic spinal cord injury: A case series and technical note. Surg. Neurol. Int..

[109] Gorgey A.S., Goldsmith J., Alazzam A., Trainer R. (2023). Effects of percutaneously-implanted epidural stimulation on cardiovascular autonomic function and spasticity after complete spinal cord injury: A case report. Front. Neurosci..

[110] Gorgey A.S., Trainer R., Sutor T.W., Goldsmith J.A., Alazzam A., Goetz L.L., Lester D., Lavis T.D. (2023). A case study of percutaneous epidural stimulation to enable motor control in two men after spinal cord injury. Nat. Commun..

[111] Gupta R., Johnson R., Samadani U. (2023). Recovery of volitional movement with epidural stimulation after “complete” spinal cord injury due to gunshot: A case report and literature review. Surg. Neurol. Int..

[112] Hoover C., Schuerger W., Balser D., McCracken P., Murray T.A., Morse L., Parr A., Samadani U., Netoff T.I., Darrow D.P. (2023). Neuromodulation Through Spinal Cord Stimulation Restores Ability to Voluntarily Cycle after Motor Complete Paraplegia. J. Neurotrauma.

[113] Samejima S., Shackleton C., Malik R.N., Cao K., Bohorquez A., Nightingale T.E., Sachdeva R., Krassioukov A.V. (2023). Spinal Cord Stimulation Prevents Autonomic Dysreflexia in Individuals with Spinal Cord Injury: A Case Series. J. Clin. Med..

[114] Kandhari S., Sharma D., Tomar A.K., Matis G., Lavrov I.A., Majumdar P. (2022). Epidural electrical spinal cord stimulation of the thoracic segments (T2–T5) facilitates respiratory function in patients with complete spinal cord injury. Respir. Physiol. Neurobiol..

[115] Smith A.C., Angeli C.A., Ugiliweneza B., Weber K.A., Bert R.J., Negahdar M., Mesbah S., Boakye M., Harkema S.J., Rejc E. (2022). Spinal cord imaging markers and recovery of standing with epidural stimulation in individuals with clinically motor complete spinal cord injury. Exp. Brain Res..

[116] Moshonkina T.R., Makarovski A.N., Bogacheva I.N., Scherbakova N.A., Savohin A.A., Gerasimenko Y.P. (2012). Effects of spinal cord electrical stimulation in patients with vertebrospinal pathology. Bull. Exp. Biol. Med..

[117] Lu D.C., Edgerton V.R., Modaber M., AuYong N., Morikawa E., Zdunowski S., Sarino M.E., Sarrafzadeh M., Nuwer M.R., Roy R.R. (2016). Engaging Cervical Spinal Cord Networks to Reenable Volitional Control of Hand Function in Tetraplegic Patients. Neurorehabil. Neural Repair.

[118] Minassian K., Hofstoetter U.S., Danner S.M., Mayr W., McKay W.B., Tansey K., Dimitrijevic M.R. (2013). Mechanisms of rhythm generation of the human lumbar spinal cord in response to tonic stimulation without and with step-related sensory feedback. Biomed. Tech..

[119] Katz P.G., Greenstein A., Severs S.L., Zampieri T.A., Singh Sahni K. (1991). Effect of implanted epidural stimulator on lower urinary tract function in spinal-cord-injured patients. Eur. Urol..

[120] Beck L., Veith D., Linde M., Gill M., Calvert J., Grahn P., Garlanger K., Husmann D., Lavrov I., Sayenko D. (2021). Impact of long-term epidural electrical stimulation enabled task-specific training on secondary conditions of chronic paraplegia in two humans. J. Spinal Cord Med..

[121] Herrity A.N., Aslan S.C., Ugiliweneza B., Mohamed A.Z., Hubscher C.H., Harkema S.J. (2021). Improvements in Bladder Function Following Activity-Based Recovery Training with Epidural Stimulation after Chronic Spinal Cord Injury. Front. Syst. Neurosci..

[122] Terson de Paleville D.G.L., Harkema S.J., Angeli C.A. (2019). Epidural stimulation with locomotor training improves body composition in individuals with cervical or upper thoracic motor complete spinal cord injury: A series of case studies. J. Spinal Cord Med..

[123] DiMarco A.F., Geertman R.T., Tabbaa K., Nemunaitis G.A., Kowalski K.E. (2021). Effects of Lower Thoracic Spinal Cord Stimulation on Bowel Management in Individuals with Spinal Cord Injury. Arch. Phys. Med. Rehabil..

[124] Burns P.B., Rohrich R.J., Chung K.C. (2011). The levels of evidence and their role in evidence-based medicine. Plast. Reconstr. Surg..

[125] Walter M., Lee A.H.X., Kavanagh A., Phillips A.A., Krassioukov A.V. (2018). Epidural Spinal Cord Stimulation Acutely Modulates Lower Urinary Tract and Bowel Function Following Spinal Cord Injury: A Case Report. Front. Physiol..

[126] Bolash R., Creamer M., Rauck R., Vahedifar P., Calodney A., Fox I., Ozaktay C., Vanquathem N. (2022). Multi-waveform Spinal Cord Stimulation with High Frequency Electromagnetic Coupled (HF-EMC) Powered Implanted Electrode Array and Receiver for the Treatment of Chronic Back and Leg Pain (SURF Study). Pain Physician.

[127] Labaran L., Jain N., Puvanesarajah V., Jain A., Buchholz A.L., Hassanzadeh H. (2020). A Retrospective Database Review of the Indications, Complications, and Incidence of Subsequent Spine Surgery in 12,297 Spinal Cord Stimulator Patients. Neuromodulation.

[128] Deer T.R., Mekhail N., Provenzano D., Pope J., Krames E., Thomson S., Raso L., Burton A., DeAndres J., Buchser E. (2014). Neuromodulation Appropriateness Consensus Committee. The appropriate use of neurostimulation: Avoidance and treatment of complications of neurostimulation therapies for the treatment of chronic pain. Neuromodulation Appropriateness Consensus Committee. Neuromodulation.

[129] Herrity A.N., Williams C.S., Angeli C.A., Harkema S.J., Hubscher C.H. (2018). Lumbosacral spinal cord epidural stimulation improves voiding function after human spinal cord injury. Sci. Rep..

[130] Nightingale T.E., Walter M., Williams A.M.M., Lam T., Krassioukov A.V. (2019). Ergogenic effects of an epidural neuroprosthesis in one individual with spinal cord injury. Neurology.

[131] Harkema S.J., Legg Ditterline B., Wang S., Aslan S., Angeli C.A., Ovechkin A., Hirsch G.A. (2018). Epidural Spinal Cord Stimulation Training and Sustained Recovery of Cardiovascular Function in Individuals with Chronic Cervical Spinal Cord Injury. JAMA Neurol..

[132] Jiang S.D., Dai L.Y., Jiang L.S. (2006). Osteoporosis after spinal cord injury. Osteoporos. Int..

[133] Battaglino R.A., Lazzari A.A., Garshick E., Morse L.R. (2012). Spinal cord injury-induced osteoporosis: Pathogenesis and emerging therapies. Curr. Osteoporos. Rep..

[134] Jilge B., Minassian K., Rattay F., Pinter M.M., Gerstenbrand F., Binder H., Dimitrijevic M.R. (2004). Initiating extension of the lower limbs in subjects with complete spinal cord injury by epidural lumbar cord stimulation. Exp. Brain Res..

[135] Ganley K.J., Willis W.T., Carhart M.R., He J., Herman R.M. (2005). Epidural spinal cord stimulation improves locomotor performance in low ASIA C, wheelchair-dependent, spinal cord-injured individuals: Insights from metabolic response. Top. Spinal Cord Inj. Rehabilit..

[136] Minassian K., Persy I., Rattay F., Pinter M.M., Kern H., Dimitrijevic M.R. (2007). Human lumbar cord circuitries can be activated by extrinsic tonic input to generate locomotor-like activity. Hum. Mov. Sci..

[137] Danner S.M., Hofstoetter U.S., Freundl B., Binder H., Mayr W., Rattay F., Minassian K. (2015). Human spinal locomotor control is based on flexibly organized burst generators. Brain.

[138] Hofstoetter U.S., Danner S.M., Freundl B., Binder H., Mayr W., Rattay F., Minassian K. (2015). Periodic modulation of repetitively elicited monosynaptic reflexes of the human lumbosacral spinal cord. J. Neurophysiol..

[139] Grahn P.J., Lavrov I.A., Sayenko D.G., Van Straaten M.G., Gill M.L., Strommen J.A., Calvert J.S., Drubach D.I., Beck L.A., Linde M.B. (2017). Enabling Task-Specific Volitional Motor Functions via Spinal Cord Neuromodulation in a Human with Paraplegia. Mayo Clin. Proc..

[140] Aslan S.C., Legg Ditterline B.E., Park M.C., Angeli C.A., Rejc E., Chen Y., Ovechkin A.V., Krassioukov A., Harkema S.J. (2018). Epidural Spinal Cord Stimulation of Lumbosacral Networks Modulates Arterial Blood Pressure in Individuals with Spinal Cord Injury-Induced Cardiovascular Deficits. Front. Physiol..

[141] Gill M.L., Linde M.B., Hale R.F., Lopez C., Fautsch K.J., Calvert J.S., Veith D.D., Beck L.A., Garlanger K.L., Sayenko D.G. (2021). Alterations of Spinal Epidural Stimulation-Enabled Stepping by Descending Intentional Motor Commands and Proprioceptive Inputs in Humans with Spinal Cord Injury. Front. Syst. Neurosci..

[142] Herrity A.N., Aslan S.C., Mesbah S., Siu R., Kalvakuri K., Ugiliweneza B., Mohamed A., Hubscher C.H., Harkema S.J. (2022). Targeting bladder function with network-specific epidural stimulation after chronic spinal cord injury. Sci. Rep..

[143] Kandhari S., Sharma D., Samuel S., Sharma G., Majumdar P., Edgerton V.R., Gad P. (2022). Epidural Spinal Stimulation Enables Global Sensorimotor and Autonomic Function Recovery after Complete Paralysis: 1st Study from India. IEEE Trans. Neural Syst. Rehabil. Eng..

[144] Sterne J.A., Hernán M.A., Reeves B.C., Savović J., Berkman N.D., Viswanathan M., Henry D., Altman D.G., Ansari M.T., Boutron I. (2016). ROBINS-I: A tool for assessing risk of bias in non-randomised studies of interventions. BMJ.

