# Supplementary material for: Epidural Spinal Cord Stimulation for Spinal Cord Injury in Humans: A Systematic Review
Source: J Clin Med. 2024 Feb 14;13(4):1090. doi: 10.3390/jcm13041090 (PMC10889415; doi:10.3390/jcm13041090)
Supplement: Supplementary file 1 [file jcm-13-01090-s001.zip › jcm-2866864-supplementary.pdf]

Supplementary

Table 1. ROBINS-I Risk of Bias Analysis[1].

	Bias due to Confounding	Bias in Selection of participants	Bias in Classification of interventions	Bias due to deviations from intended interventions	Bias due to missing data	Bias in measurement of outcomes	Bias in selection of reported result
Barolat (1986) [108]	Serious	Serious	Serious	Low	Low	Serious	Moderate
Huang (2006) [33]	Moderate	Serious	Moderate	Low	Low	Serious	Low
Monshonkina (2012) [117]	Serious	Moderate	Serious	Serious	Low	Serious	Moderate
Minassian (2013) [119]	Serious	Serious	Serious	Low	Low	Serious	Moderate
Lu (2016) [118]	Serious	Moderate	Moderate	Low	Low	Serious	Low
Wagner (2018) [42]	Moderate	Moderate	Moderate	Moderate	Low	Serious	Low
Calvert (2019) [43]	Moderate	Moderate	Moderate	Moderate	Low	Serious	Low
Darrow (2019) [45]	Serious	Moderate	Moderate	Low	Low	Serious	Low
Gorgey (2020) [46]	Serious	Serious	Serious	Moderate	Moderate	Serious	Moderate
Calvert (2021) [48]	Serious	Moderate	Serious	Low	Low	Serious	Low
Linde (2021) [50]	Serious	Moderate	Moderate	Low	Low	Serious	Low
Gorgey (2022) [52]	Serious	Serious	Serious	Low	Low	Serious	Moderate
Kandhari (2022) [144]	Serious	Moderate	Moderate	Low	Low	Serious	Low
Rowald (2022) [53]	Serious	Serious	Moderate	Low	Low	Serious	Low
Smith (2022) [116]	Serious	Serious	Moderate	Low	Low	Serious	Low
Boakye (2023) [109]	Serious	Moderate	Moderate	Low	Low	Serious	Low
Gorgey (2023) A [110]	Serious	Serious	Serious	Low	Low	Serious	Moderate
Gupta (2023) [112]	Serious	Serious	Serious	Low	Low	Serious	Moderate
Hoover (2023) [113]	Serious	Moderate	Moderate	Low	Low	Serious	Low

Reference

Sterne, J.A.; Hernán, M.A.; Reeves, B.C.; Savović, J.; Berkman, N.D.; Viswanathan, M.; Henry, D.; Altman, D.G.; Ansari, M.T.; Boutron, I.; et al. ROBINS-I: A tool for assessing risk of bias in non-randomised studies of interventions. *BMJ* **2016**, *55*, i4919.
